# Functional eubacteria species along with trans-domain gut inhabitants favour dysgenic diversity in oxalate stone disease

**DOI:** 10.1038/s41598-018-33773-5

**Published:** 2018-11-09

**Authors:** Mangesh V. Suryavanshi, Shrikant S. Bhute, Rahul P. Gune, Yogesh S. Shouche

**Affiliations:** 1 National Centre for Microbial Resource, National Centre for Cell Science, Central Tower, Sai Trinity Building Garware Circle, Sutarwadi, Pashan Pune, 411021 (M.S.) India; 2 Yenepoya Reseach Centre, Yenepoya University, Mangalore, 575018 (K.S.) India; 3 School of Life Sciences, University of Nevada, Las Vegas, Nevada-89154 USA; 4 Department of Urology, RCSM Govt. Medical College, CPR Hospital Compound, Bhausingji Rd, Kolhapur, 416002 (M.S.) India

**Keywords:** Microbial ecology, Microbial ecology

## Abstract

Analyses across all three domains of life are necessary to advance our understanding of taxonomic dysbiosis in human diseases. In the present study, we assessed gut microbiota (eubacteria, archaea, and eukaryotes) of recurrent oxalate kidney stone suffers to explore the extent of trans-domain and functional species dysbiosis inside the gut. Trans-domain taxonomic composition, active oxalate metabolizer and butyrate-producing diversity were explored by utilizing *frc-, but-*, and *buk-* functional gene amplicon analysis. Operational taxonomic units (OTUs) level analyses confound with the observation that dysbiosis in gut microbiota is not just limited to eubacteria species, but also to other domains like archaea and eukaryotes. We found that some of healthy eubacterial population retained together with *Oxalobacter formigenes* and *Lactobacillus plantarum* colonization in disease condition (p < 0.001 & FDR = 0.05). Interestingly, trans-domain species diversity has been less shared and dysgenic taxa augmentation was found to be higher. Oxalate metabolizing bacterial species (OMBS) and butyrate-producing eubacteria species were found to be decreased in *Oxalobacter* non-colonizers; and *Prevotella* and *Ruminococcus* species which may contribute to oxalate metabolism and butyrate synthesis as well. Our study underscores fact that microbial dysbiosis is not limited to eubacteria only hence suggest the necessity of the trans-domain surveillance in metabolic diseases for intervention studies.

## Introduction

The human gut microbiome is considered as an effective metabolic organ; several host vital metabolite dependencies have been systematically incorporated and disparagingly executed within gut by it. In fact, causes and consequences of many metabolic disorders have been rationalized using high correlation with specific gut inhabitants. Dysbiosis has been usually attributed to eubacteria as previously reported in literature. Variations in the eubacterial community referred to as dysbiosis; disturbs the host health status and is found *actus-reus* principally in intestinal syndromes. Upon dissecting non-intestinal metabolic disorders like obesity^1^, cardiovascular disease^2^ and chronic kidney disease^3^, it was understood that dysbiosis in gut eubacteria has partial verdict and indicates towards the direction for interventions in the diagnosis, treatment, and probable prevention. Most of the microbiome components have originated from the eubacteria and are diverse in genes, genomes, and their products in gut ecology^4^. However, other components remain elusive regarding knowledge of their diversity and role in host health.

Trans-domain gut inhabitants include eubacteria, archaea, eukaryotes and their diversity are the key players in gut ecology. Trans-domain diversity inside the human gut is not yet fully explored in healthy state as well as in impaired health condition. In some reports generated on diabetes; *Methanobacter* (archaea) and *Aspergillus* species (filamentous fungi) were found to be prevalent in the diabetic state^5^.

Using high throughput sequencing methods, the variations in healthy gut flora were observed and disease associated bacteria were traced universally. The persistent colonization of unique gut microflora involved in non-intestinal metabolic disorders has been correlated with host health conditions. Moreover, disease associated flora for kidney stone disease was manifested and re-enlisted specifying signature flora in canines^6^, and humans by prognosticative ways^7^. The panoptic power of high throughput sequencing methods was confined to describe the species level taxonomic features of gut bacteria as it has limitations of short-read length as output. Prognosticative detection of disease associated flora is still elusive mainly due to the lack of the species-level taxonomic assignments. Apparently, defining the role of species of the gut inhabitant, along with qPCR-based species-specific detection of *Oxalobacter formigenes* have been indispensable in kidney stone endurers^8^ and only the metagenomic sequencing approach has been found productive^9,10^.

Functional metagenome strategy has proven to support the hypothesis related to the metabolic capacities and its diverse origin in the health status. Functional capacities of the eubacteria from the previous observations^11^, gave clues about the dynamics observed in the hyperoxaluria condition due to their activities. Here, we are reporting a comprehensive catalog of functional gene diversity using functional metagenomic library. We used the functional genes such as *frc-*gene to decipher OMBS diversity and *but-* and *buk-*gene for butyrate-producing bacterial species (SCFA producer) diversity. Comprehensive reports on species level diversity in gut inhabitants and kidney stone associated dysbiosis in human subjects were lacking. In the present study, we focused on assessment of species-level differences between the compositions of the intestinal flora in individuals with recurrent kidney stone to that of healthy individuals based on 16S rRNA gene traditional clone libraries for the eubacteria, 18S rRNA gene for microeukaryotes and ITS region for the fungal species diversity analysis.

## Results

Posited emergence of exploitation of use of the indigenous gut bacteria for recurrent episode repression was recommended^12^. The colonization of *Oxalobacter formigenes*, a known oxalotrophic bacterium was directly linked to the disease prevalence^8^. In addition to the case-control study for recurrent kidney stone disease^11^, included healthy control study subjects to check whether that certain other healthy gut associated bacterial species may also dwell with the recurrent oxalate stone episodes. The comparatives in healthy subjects (HLT) and kidney stone disease subjects (KSD) (Supplementary Table S1) has been explained by using amplification and sequencing of functional genes and trans-domain molecular marker genes (Table 1).

**Table 1:** Eubacterial species enriched in KSD subjects and their possible involvement in health impairments.

Bacterial species	Explanations for Involvement in various diseases	
	Microbiota associated	Pathological detection
*Lactobacillus mucosae*	NA	Dental caries and Dental lesions^51^
*Olsenella umbonata*	NA	Dental caries and Dental lesions^52^
*Collinsella aerofaciens*	Inflammatory Bowel Disease^53^	Colitogenic bacteria in inflammation^54^
*Streptococcus sanguinis*	Inflammatory Bowel Disease^53^	Colitogenic bacteria in inflammation^54^
*Eubacterium siraeum*	Behcet’s disease^55^, exhibit the highest affinities for Candida albicans^56^	Colitogenic bacteria in inflammation^57^
*Streptococcus lutetiensis*	NA	Children diarrhoea^58^
*Turicibacter sanguinis*	Children with Pervasive Developmental Disorder Not Otherwise Specified (PDD-NOS) and autism (AD)^59^	NA
*Veillonella dispar*	Patients with hepatitis B liver cirrhosis^60^	Endocarditis^61^
*Comamonas testosteroni*	NA	Bacteraemia^62^, Acute Appendicitis^63^
*Escherichia fergusonii*	NA	Bacteraemia^64^, Pneumonial pathogenesis^65^
*Klebsiella pneumoniae*	NA	Pneumonial pathogenesis^66^
*Succinivibrio dextrinosolvens*	Associated with cancer development^67^	NA
*Megamonas funiformis*	Effects on Immunity and Disease^68^	NA
*Catenibacterium mitsuokai*	Effects on Immunity and Disease^68^	NA

^*^NA = Not any literature found.

### Comparison of eubacterial species and its functional diversity in HLT and KSD

**Figure 1:**
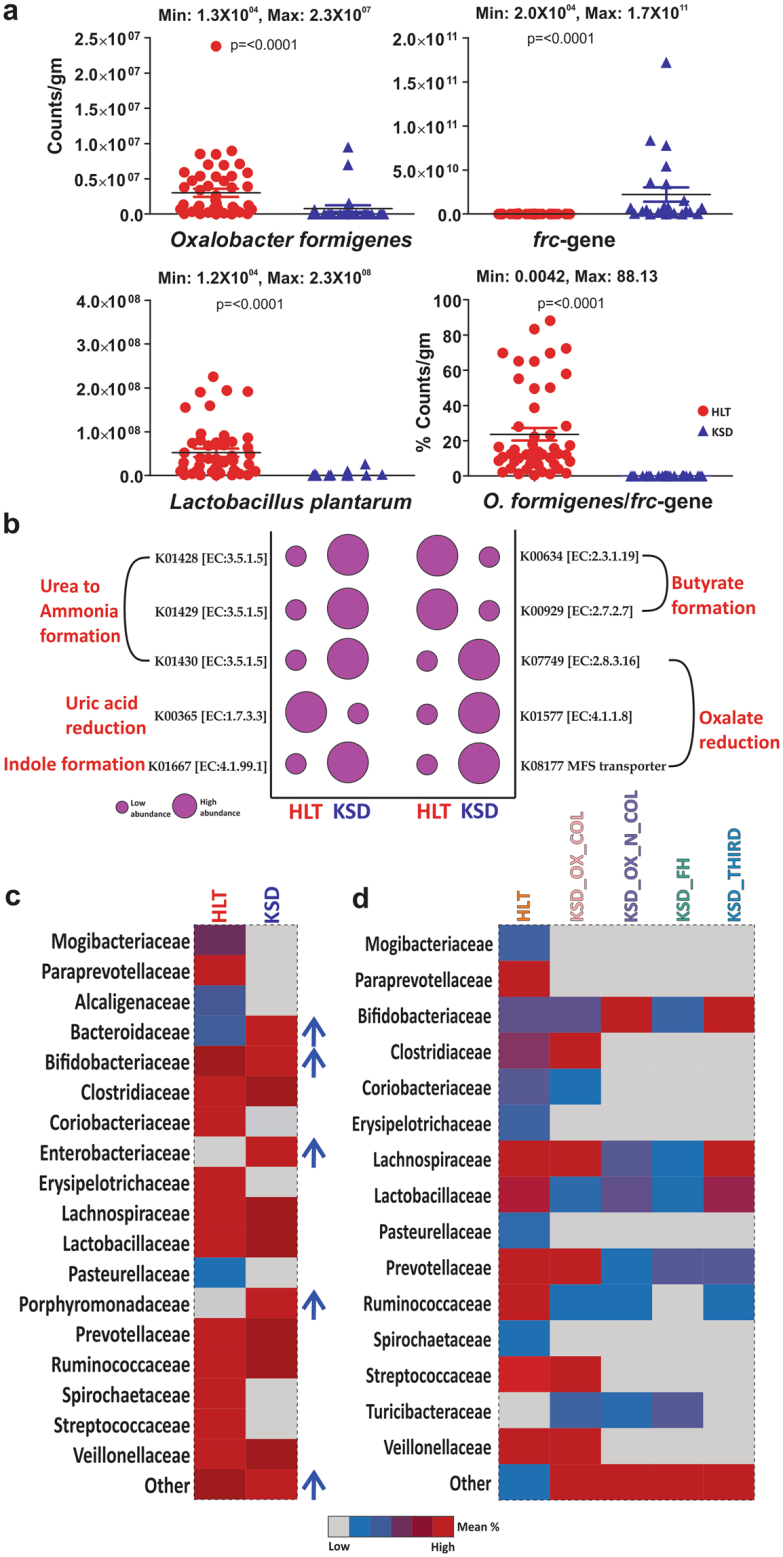
Comparatives in eubacterial colonization status of HLT and KSD subjects. (**a**) qPCR based quantitative estimation of *Oxalobacter formigenes*, total OMBS diversity (through *frc-*gene) and *Lactobacillus plantarum* in HLT and KSD (n = 72). (**b**) Evaluation of specific metabolism capacities of eubacteria, and respective KEGG Orthology for the enzymes involved in metabolic phenotype according to the predicted metagenome observed in HLT and KSD. (**c**) Heat map showing family level differences in HLT and different groups in KSD subjects. The data used to generate these graphs has been partially adopted from Suryavanshi *et al*.^11^.

The oxalate metabolizing bacteria accounted for the highest at 88.13% among the enriched in KSD and found to be unique feature of oxalate kidney stone patients (KSD) (Fig. 1a). The qPCR results show increased abundance of *frc*-gene in KSD in comparison to the HLT. However, the *Oxalobacter formigenes* and *Lactobacillus plantarum* abundance was decreased in the KSD group. We had already demonstrated the bacterial dysbiosis and enrichment of unique OMBS in subpopulation of this cohort, we reported the bacterial dysbiosis and enrichment of unique OMBS^11^. We found that the colonization of *Oxalobacter* in the KSD subjects retains the 118 out of 785 OTUs detected (Kruskal-Wallis test, p <= 0.001 & FDR = 0.05), reflecting the 20.08% of HLT group flora (Supplementary File S2). Such keen observation depicts that the retention of HLT microbiome could be achieved by the *Oxalobacter formigenes* and *Lactobacillus plantarum*.

The metagenomic imputation demonstrated the differential abundance of genes involved in oxalate metabolism in KSD (Wilcoxon sum rank test, p =< 0.005) (Fig. 1b). We found out that certain families are characteristics of disease in the KSD subjects and in the different subjects. Certain bacterial families like Bacteriodaceae, Enterobacteraceae, and Porphyromonadaceae were found enriched and Mogibacteriaceae, Paraprevotellaceae, Alcaligenaceae, Pasterallaceae, Spirochaetaceae and Streptococcaceae were depleted in KSD (Welch’s t-test, p =< 0.001 & FDR = 0.05) group (Fig. 1c,d). As reported earlier^13^; applying the hypergeometric test function (p <= 0.001), we found that such differential bacterial families are present and have their own role in KSD not by chance mechanism.

**Figure 2:**
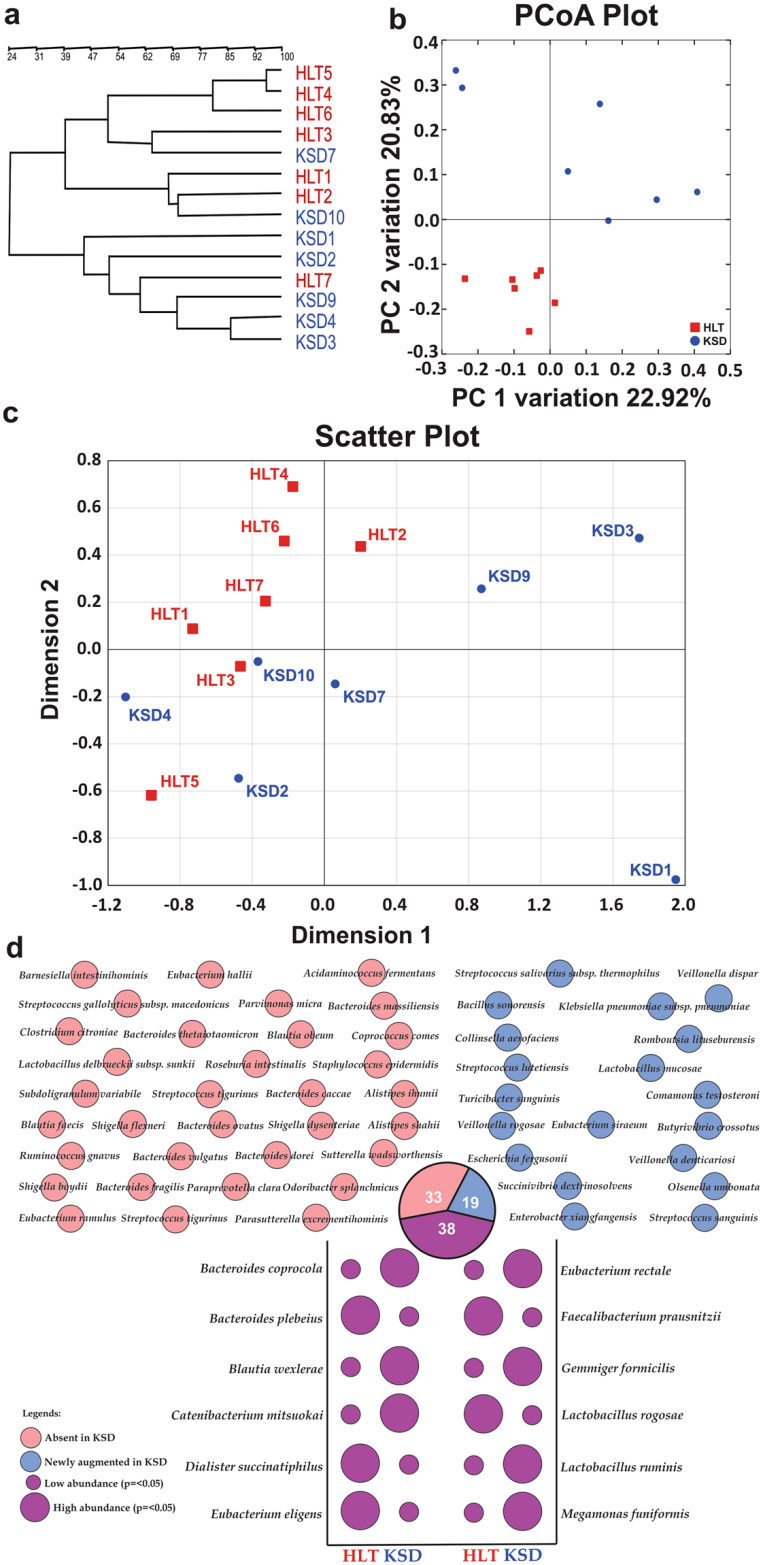
Heat map showing the status of functional eubacteria in different groups in tested subjects. (**a**) heat map for total OMBS diversity players observed by *frc-*gene sequence analysis, and (**b**,**c**) reflects the Butyrate (known-SCFA) producer observed by *but-* and *buk-*gene sequence analysis respectively. Only presence-absence data represented here whereas the OMBS diversity data has been adopted from Suryavanshi *et al*.^11^.

In connection with functional capacities, surveillance of functional eubacteria was accessed through the *frc-, but-*, and *buk-*genes in the subpopulation (Supplementary Table S3). The species level diversity of functional genes which are unique present in comparative, reported in (Fig. 2). Eleven bacterial species accessed through *frc-*gene were found to be completely absent in the KSD subpopulations in comparison to the healthy subjects. The functional gene diversity pattern of KSD was found dislodged to the HLT. Similarly, 23 bacterial species accessed through *buk-*gene were not found to be present in the KSD group. The functional group assessed through *buk*-gene revealed that only four bacterial species found in the healthy subjects diminished completely in the KSD subjects.

### Deducing the change in eubacteria species through clone library generation approach in KSD

**Figure 3:**
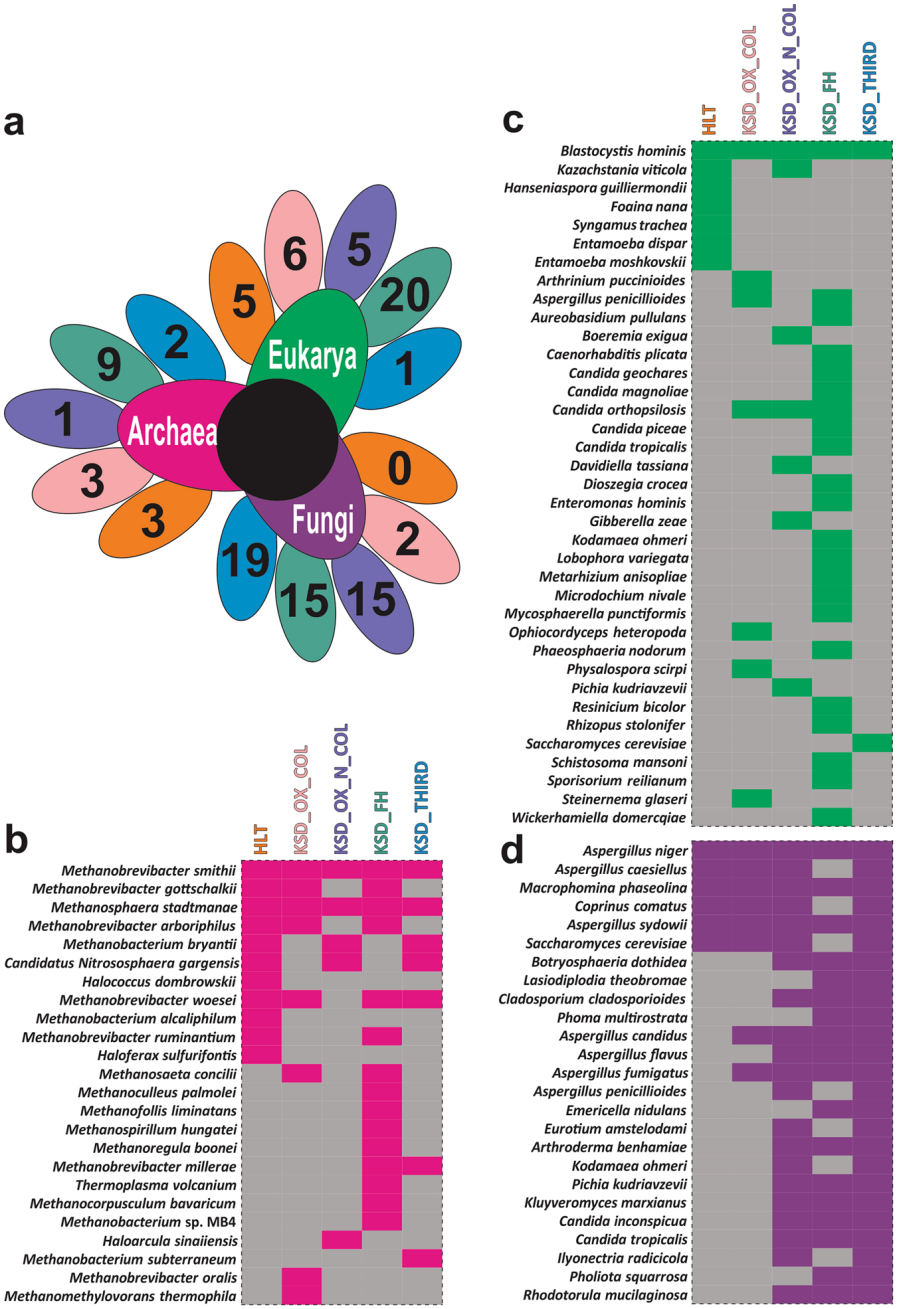
Compositional differences in eubacteria diversity in study subpopulations (HLT = 07 and KSD = 07). Clustering pattern of tested subpopulation through (**a**). PCR-DGGE fingerprint analysis in UPGMA algorithm, (**b**). PCoA plot and (**c**). IKF values on scattered plot derived from K-Shuff algorithm. (**d**) Species level diversity differences observed in HLT and KSD subjects, whereas shared species with significant difference (Welch’s test p <= 0.05) were annotated in bubble plot.

For the bacterial species diversity characterization, we obtained 16S rRNA gene clones and PCR-DGGE fingerprinting patterns of some randomly selected subjects. Total 6400 clones were randomly picked and sequenced from fourteen fecal samples from which 5057 good quality sequences of more than 750 base pairs with an average of 361 clones per sample were obtained. Measures of alpha diversity such as Cho1, Shannon and Simpson indices showed significant decrease in overall diversity in KSD subjects (Supplementary Table S4). All the gut communities contained sequences from 7 bacterial phyla (Firmicutes, Bacteroidetes, Proteobacteria, Cyanobacteria, Tenericutes and Thermi). Denaturant gradient gel electrophoresis of eubacterial 16S rRNA gene revealed differential fingerprinting pattern for KSD and HLT individuals suggestive of bacterial community shift as indicated by similarity profile of DGGE band pattern for all the subjects (Fig. 3a). We also analyzed the OTU abundance from each sample which corresponded to 23 different families. The bacterial families were differentially abundant in the KSD subjects (Supplementary Fig. 1). PCoA analysis of unweighted UniFrac distance matrix revealed distinct clustering of HLT subjects than discrete spread of KSD subjects (Fig. 3b). Clustering of IKF values obtained from K-Shuff revealed grouping of all HLT subjects (Fig. 3c).

We found that 833 and 90 OTUs were assigned to genus and species level identity by >95.0% and >97.0% of sequence identity cut off value respectively (Supplementary Table S5). Out of 90 bacterial species, 33 were completely depleted and 19 were newly augmented in KSD subjects over HLT. While 12 bacterial species (p =< 0.01) were found to be differentially present in KSD subjects (Fig. 3d). These results suggest that some bacterial species were found to be absent in the recurrent oxalate stone episodes and some new bacterial species were found to be present in such conditions. All newly augmented flora were found to be mostly aerobic and facultative *Streptococcus sanguinis*, *Bacillus sonorensis*, *Comamonas testosterone*, *Klebsiella pneumoniae* subsp. *pneumoniae*, *Escherichia fergusonii* and *Veillonella denticariosi*. Deserting the differential eubacteria species in KSD, most are perpetually positive in their involvement in various health impairments and sometimes may act as etiological agents (Table 1).

### Differences in gut inhabiting archea, eukarya and fungal species in KSD

**Figure 4:**
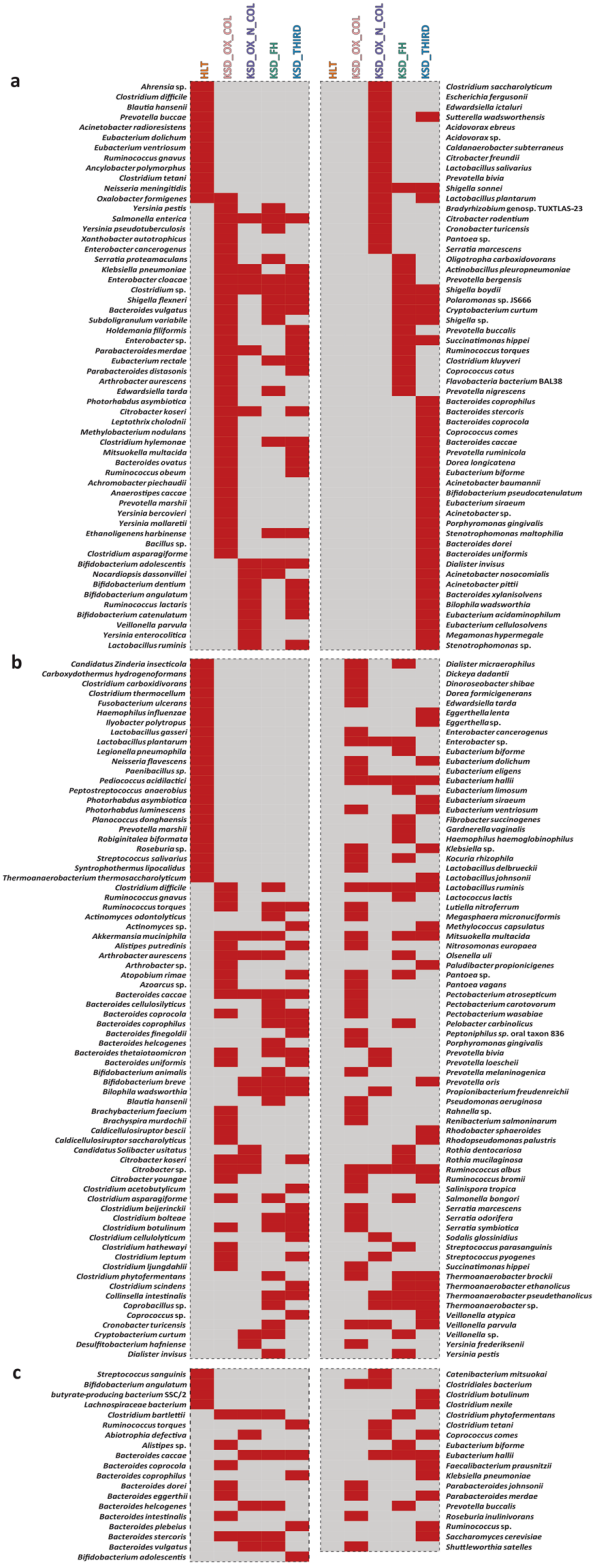
Trans-domain species diversity present in 5 groups (**a**) Diagrammatic illustration of unique Trans-domain species in each group, whereas comparison in HLT and KSD subjects only. The color of petal indicates the number of unique species either in HLT or KSD subject within 5 group. (**b**–**d**) Heatmap showing the species diversity in each five groups with pink (archaea), green (microeukaryotes) and violet (fungi) represents actual trans-domain components. Diversity was studied by the conserved genes such as 16S rRNA gene (archaea and eubacteria), 18S rRNA gene (microeukaryotes) and ITS region (fungi) sequencing. Presence and absence of gene bearing species data utilized for the representation.

**Figure 5:**
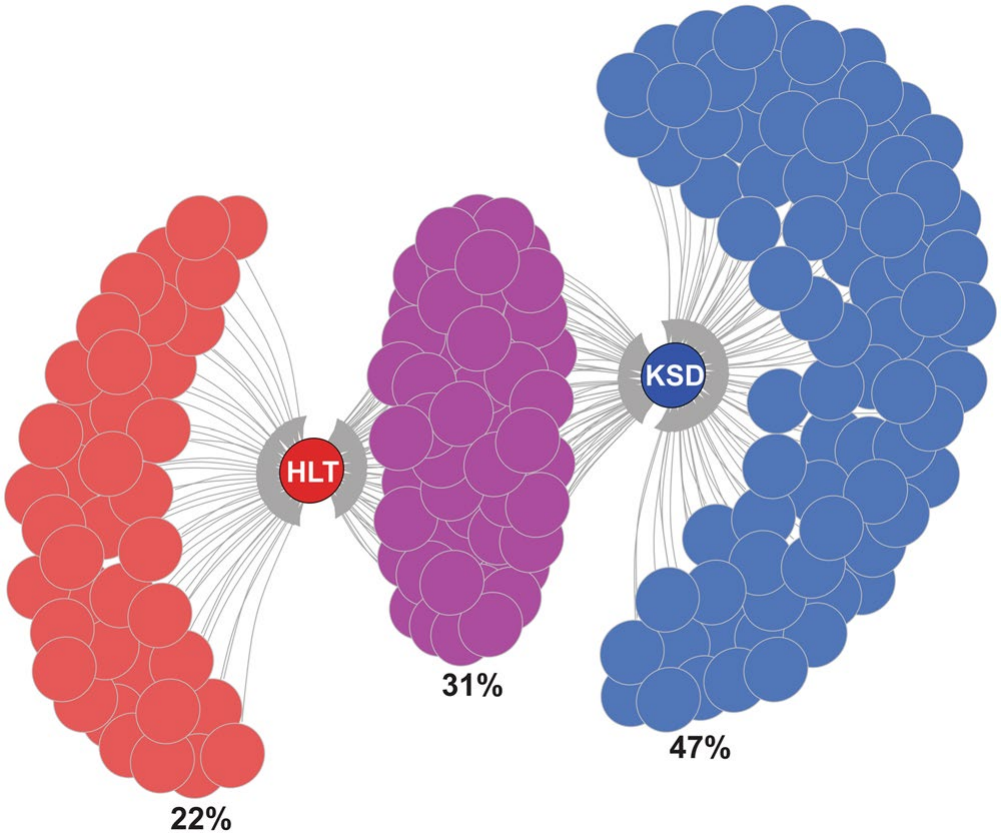
Diagrammatic illustration of Trans-domain species found in the tested subjects. An overall number of Trans-domain species (eubacteria, archaea, fungi, and microeukaryotes) in HLT and KSD subjects were demonstrated and presented only on presence or absence data through igraph. The Tran-domain species which were uniquely distributed in either HLT or KSD subjects are represented by red (HLT), blue (KSD) and violet color for common species.

As far as the trans-domains are concerned, eubacteria along with archaea, eukaryotes and fungi are the gut inhabitants and major diversity players (Supplementary Table S6). We found that the 11 members of archaea, 7 of eukarya and 6 fungal ubiquitous species of HLT group were absent in KSD subjects (Fig. 4). The co-occurrence pattern illustrated a comprehensive change in the microbial community of the KSD subjects in comparison to the healthy individuals (Fig. 5).

## Discussion

Kidney stones are believed to be multi-factorial diseases and are predominantly found in male than in female^14^. Although, the oxalate stones in urinary system are perceived as a part of hyperoxaluria in many individuals may with recurrent episodes, and sometimes leading to chronic disease^15^. In last two decades, satisfactory regime for diagnosis and treatment of recurrent kidney stones focused on the factors affecting handling of oxalate by the intestine as a part of homeostasis driven by the host^16^ and rather the structure and composition of gut microbial communities inside the gut^17^. Dysbiosis associated with pathogenesis of the kidney and the large intestine microbiome would be the bi-directional operative axis for its proper functioning. In connection with altered microflora with respect to chronic kidney disease^18,19^, the specific reports on alteration in gut microbiome associated with kidney stones of human subjects and their metabolic potentials are little scanty. We present this study as an attempt to elucidate the changes in the trans-domain microbial species including eubacteria, archaea, fungi and micro-eukaryotes, and their function in lieu of kidney stone disease.

### Eubacterial family and their functional alterations are characteristics of oxalate stone disease

Oxalate influences the colonic microbial metabolism whereas; microbial-related metabolites are speculated to be involved in the progression of the kidney disease. In the present study, we applied two culture-independent approaches namely 16S rRNA clone library and PCR-DGGE to reveal bacterial community structure of gut flora in KSD subjects. Qualitative and quantitation perturbations in functional eubacteria species also were found.

Major players like *Oxalobacter formigenes* and *Lactobacillus plantarum* species are the key eubacteria in Indian healthy gut and their quantitative values provides the reference material in colonization status even in oxalate kidney stone disease. *Lactobacillus plantarum* species has been reported from the healthy gut flora in the same population^20^, and here we found out negative correlation with hyperoxaluria condition even high with their oxalate tolerance capacity evaluated previously.

From clustering pattern based on PCR-DGGE and PCoA plot based on unweighted UniFrac we observed that KSD subjects are dispersed as compared to HLT subjects. We speculate that this may be due to more different OTU composition of KSD than HLT and among them. Inter-individual difference among the KSD subjects could be because of recurrent episodes of kidney stones and different number of stones.

Our findings on human subjects showing increased abundance of Firmicutes and decreased abundance of Bacteroidetes are in agreement with the previous studies relating to the gut microflora and the oxalate stones in humans^7,21^ and even in the dogs^6^. However, at the genus level various distinctions are observed confirming the fact that the gut microbiota varies according to the host type^22^. We observed dominance of genera such as *Collinsella* and reduction in genera like *Bacteroides*, *Deinococcus and Sutterella* in KSD group. *Collinsella* is known to be involved in deconjugation of bile acids^23^ and is also associated with disease like symptomatic atherosclerosis^24^. Thus, high abundance of *Collinsella* in the gut microbiota may be an indicator of a disturbed gut. Bacterial phylotypes of *Bacteroides* genera are potentially involved in glycan synthesis^25^. Highly robust bacteria such as *Deinococcus* have been shown to be adapted for human gut colonization^26^ and members of *Sutterella* have been found to be linked with autism disease in children^27^.

The change in the gut microbial community structure of KSD patients in comparison with the healthy subjects could be attributed to the diet patterns. Strict dietary restrictions in advanced CKD for high-fiber products could affect the makeup and/or metabolism of the gut flora^3^.

### Alterations are dislodged to the functional eubacteria species diversity

On the other hand, species level dysbiosis was found in disease conditions whereas, certain bacterial species which were involved in other diseases were augmented. Lactic acid fermenting-mucosa associated bacteria, *Olsenella umbonata* has ability to produce 4-methylphenol (p-cresol) from p-hydroxyphenylacetic acid^28^. Absolute findings of novel bacterial species in human gut are leading towards greater exploration of gut microflora then we have ever speculated^29^. Some of species like *Eubacterium siraeum* has been reported for biosynthesis of conjugated linoleic acid^30^ and *Klebsiella pneumoniae* have a phospholipase D family protein as a novel virulence factor^31^.

For reduction in glycans and complex carbohydrates helping bacteria were depleted *Bacteroides plebeius*^32^. Propionate production within the human gut microbiota was reported including *Dialister succinatiphilus*^33^. *Eubacterium eligens* has known for dietary fibers production by pectin^34^. Downshift pH 5.5 resulted in *Faecalibacterium prausnitzii* lowering and underlying atopic dermatitis^35^. Variety of carbohydrates including the inulin, pectin can be metabolized by *Faecalibacterium* and have efficient processing ability of D-glucosamine and N-acetyl-d-glucosamine^36^. *Faecalibacterium prausnitzii* is known anti-inflammatory commensal flora^37^ which have the ability to grow at oxic–anoxic interphases^38^. This fact suggests that either hyperoxaluric condition may be responsible for enrichment of some oxalate utilizing bacterial genera that are not observed in healthy subjects; or due to the disturbed microflora inside the gut may impart the hyperoxaluric condition to the host are still unclear.

Our PICRUSt analysis revealed several important findings of gut microbiome in relation to calcium oxalate stone formation. Inositol, which is also known as phytate seems to have important role in preventing calcium oxalate stone formation. Levels of inositol was found to be significantly reduced in people with calcium oxalate stone formers^39^ and in rat models phytate has been shown to have inhibitory role on deposition of calcium oxalate stones^40^. Previous study has shown dissolution effect of lysine on calcium oxalate by considerably lowering the super-saturation of the urine^41^. Similarly, there are reports showing reduced levels of tyrosine in stone formers compared to control subjects^42^. Interestingly, our results showed significant degradation of lysine and metabolism of tyrosine by gut microbiota in KSD subjects suggesting that effective concentration of lysine and tyrosine needed for dissolution of calcium oxalate stones was never attained in these subjects, thus chances of recurrent stone formation are increased in these subjects. We observed that gut microbiota of KSD subjects was dominant with gene families involved in fatty acid synthesis. Fatty acid and bile acid malabsorption is a one of the risk factors for hyperoxaluria in mal-absorptive patients and low fat diet is often advocated to these patients to prevent hyperoxaluria^43^.

### Trans-Domain gut inhabitants favor the dysgenic diversity in oxalate disease

In the human gut diverse prokaryotic populations have been studied well compared with that of the eukaryotic fraction which are still underway^29,44^. The current studies on the gut resident microbial eukaryotes give clues for either beneficial or commensal. Elucidating such crucial piece of gut community to cause of several diseases might be potential treatment factor cannot be denied.

**Figure 6:**
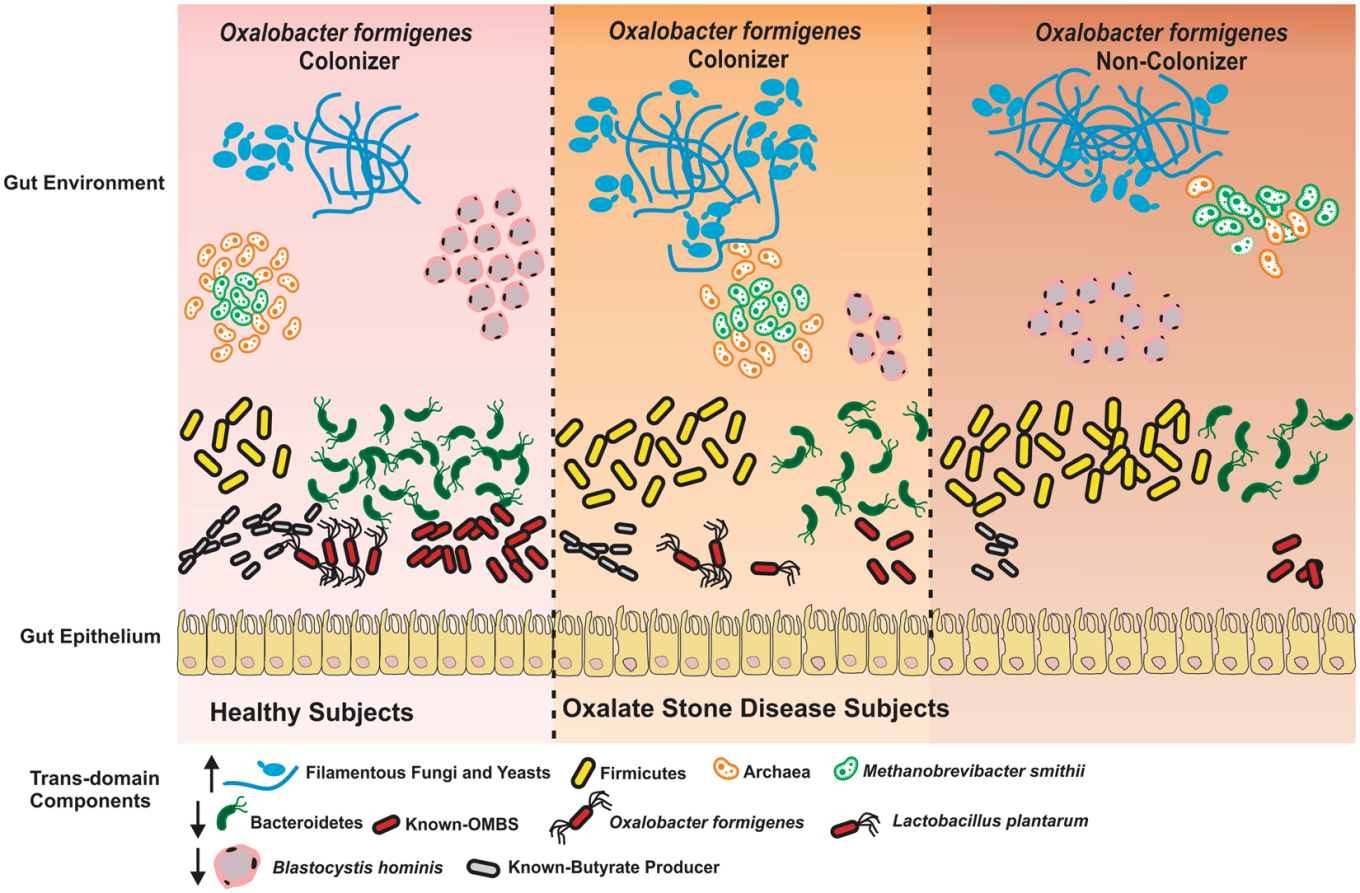
Diagrammatic illustration of actual observations in all tested subjects. Eubacterial species diversity and functional diversity was depicted using amplicon sequencing and qPCR assays. Other Trans-domain diversity was depicted using amplicon sequencing based analysis only. Arrow indicates the abundance level of trans-domain components in disease state.

Although the methanogens, species like *Methanobrevibacter smithii* and *Methanosphaera stadtmanae* have the most dominant archaeal groups inside the human gut^45,46^. The two fungal phyla Ascomycota (which includes the genera *Candida* and *Saccharomyces*) and Basidiomycota are the prominent fungal diversity^47,48^. Our observation is also in agreement with the archaeal and fungal diversity in Indian population. Previous reports have indicated that dysbiosis in the fungal population of the human gut can be associated with intestinal disease and infections. In the present study, there is an evident change in the eukaryotic, archaeal and fungal populations in KSD patients as compared with healthy individuals. The results suggest that the selection pressure on the part of the host and the microbes shapes the structure, composition, and function of not only the bacterial populations but also the trans-domain diversity. However, this is the first report indicating a direct correlation between the kidney stone disease and altered diversity of gut microflora involving archaeal and fungal participants. Despite maintaining of an ecosystem the colonization of *Oxalobacter formigenes* inside the human gut as healthy gut flora^10^ describing the withholds host health features and other core microbiome as well^9^. As their absence corresponds to enhanced level of oxalate in systemic fluid^49^, replacement therapy granted the recovery from hyperoxaluria condition in humans^50^. Demonstrating the colonization status of *Oxalobacter formigenes*, we speculate the major role of this bacterium in settlement of functional eubacteria and trans-domain species as well as in symptomatic phase of hyperoxaluria i.e. oxalate stone disease (Fig. 6). Presence of *Oxalobacter formigenes* has positively impacted on *Lactobacillus plantarum*, known butyrate producers and Bacteriodetes members’ colonization and inversely related to *Methanobrevibacter smithii*, filamentous fungi and Fermicutes members’ in this Indian population study.

In conclusion, our study reports that the dysbiosis of gut microbial communities in recurrent oxalate kidney stone sufferers are limited not just to structural but also to the active functional players. Even though our study was limited to small number of participants; the results highlight the vast diversity of oxalate metabolizing bacteria in the human gut. Metabolic features, immunological response panel and disturbed trans-domain species correlation may give a definitive picture of hyperoxaluria condition. We hope our study would provide basic clues for high throughput DNA sequencing studies linking the role of gut microbiota and more information on trans-domain species in the development of oxalate kidney stones. Subsequently it helps to formulate the microbiome based biomarkers for its recurrent episodes.

## Materials and Methods

### Study samples and data generation

Inclusion of all the recruited subjects was based on the National Centre for Cell Science’s institutional ethics committee’s approval; in accordance with declaration of Helsinki principles. A total of seventy-two subjects were enrolled with their separate written informed consent form; symptomatic kidney stone diseased (n = 24) hereafter referred to as KSD, and Healthy control (n = 48) hereafter called HLT were involved in the present study. Subpopulations from this cohort had been already explained elsewhere as a case-control study^11^. Sample collection, processing, amplicon- and functional- metagenomic data generation with subsequent analysis has been explained in Supplementary File S7.

### Raw sequence deposition

Eubacterial clone library sequences were deposited at NCBI GenBank with accession numbers KF229803-KF233412 and KF864681-KF866127. Raw sequences generated through Ion Torrent PGM in the present study including *frc-, but-*,and *buk-*gene as functional genes and Archaeal 16S rRNA gene, microeukaryotic 18S rRNA gene and fungal ITS region as trans-domain gene amplicons were deposited to SRA-NCBI under accession number SRP067039.

## Electronic supplementary material

Supplementary Tables

Supplementary File S2

Supplementary File S7
