# Author Correction: Functional eubacteria species along with trans-domain gut inhabitants favour dysgenic diversity in oxalate stone disease

**DOI:** 10.1038/s41598-019-41872-0

**Published:** 2019-05-23

**Authors:** Mangesh V. Suryavanshi, Shrikant S. Bhute, Rahul P. Gune, Yogesh S. Shouche

**Affiliations:** 1 National Centre for Microbial Resource, National Centre for Cell Science, Central Tower, Sai Trinity Building Garware Circle, Sutarwadi, Pashan Pune, 411021 (M.S.) India; 2 Yenepoya Reseach Centre, Yenepoya University, Mangalore, 575018 (K.S.) India; 3 School of Life Sciences, University of Nevada, Las Vegas, Nevada 89154 USA; 4 Department of Urology, RCSM Govt. Medical College, CPR Hospital Compound, Bhausingji Rd, Kolhapur, 416002 (M.S.) India

Correction to: *Scientific Reports* 10.1038/s41598-018-33773-5, published online 09 November 2018

This Article contains an error in the order of the Figures. Figures 2, 3 and 4 were published as Figures 4, 2, and 3 respectively. The correct Figures 2, 3 and 4 appear below as Figs 1, 2 and 3 respectively. The Figure legends are correct.

**Figure 1:**
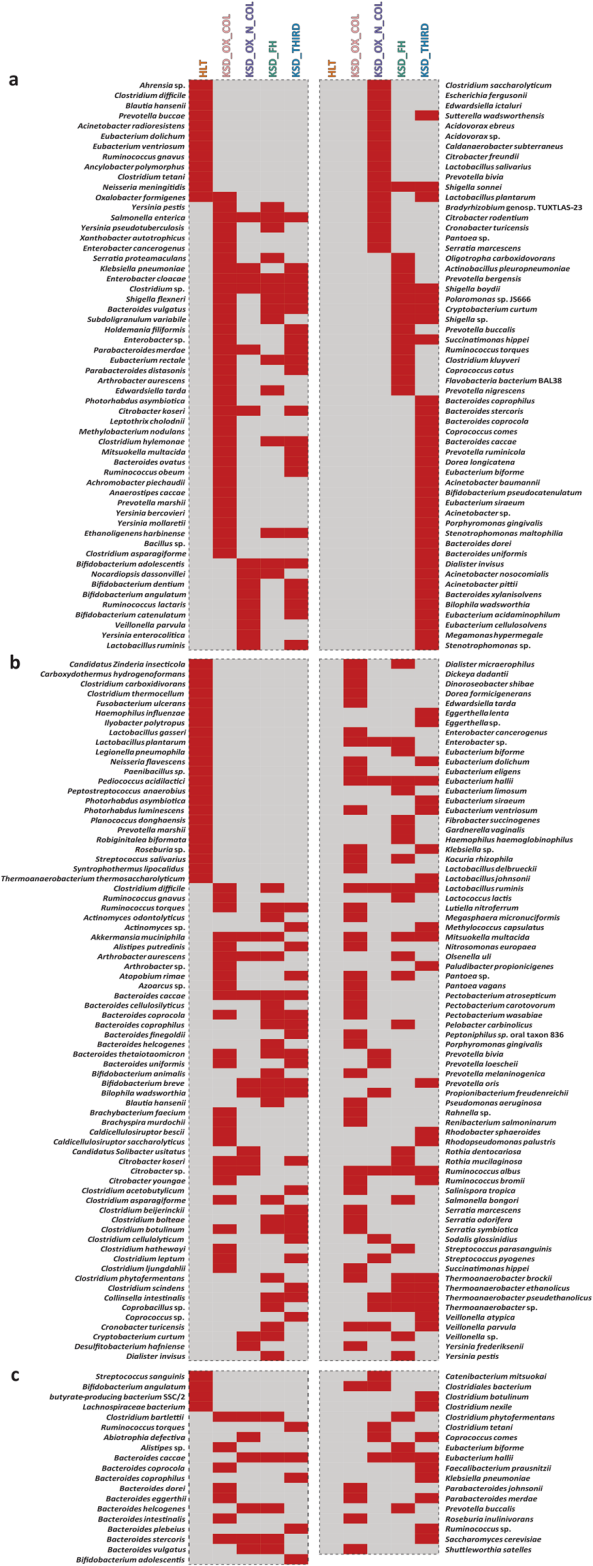
Heat map showing the status of functional eubacteria in different groups in tested subjects. (**a**) Heat map for total OMBS diversity players observed by frc-gene sequence analysis, and (**b**,**c**) reflects the Butyrate (known-SCFA) producer observed by but- and buk-gene sequence analysis respectively. Only presence-absence data represented here whereas the OMBS diversity data has been adopted from Suryavanshi *et al*.^11^.

**Figure 2:**
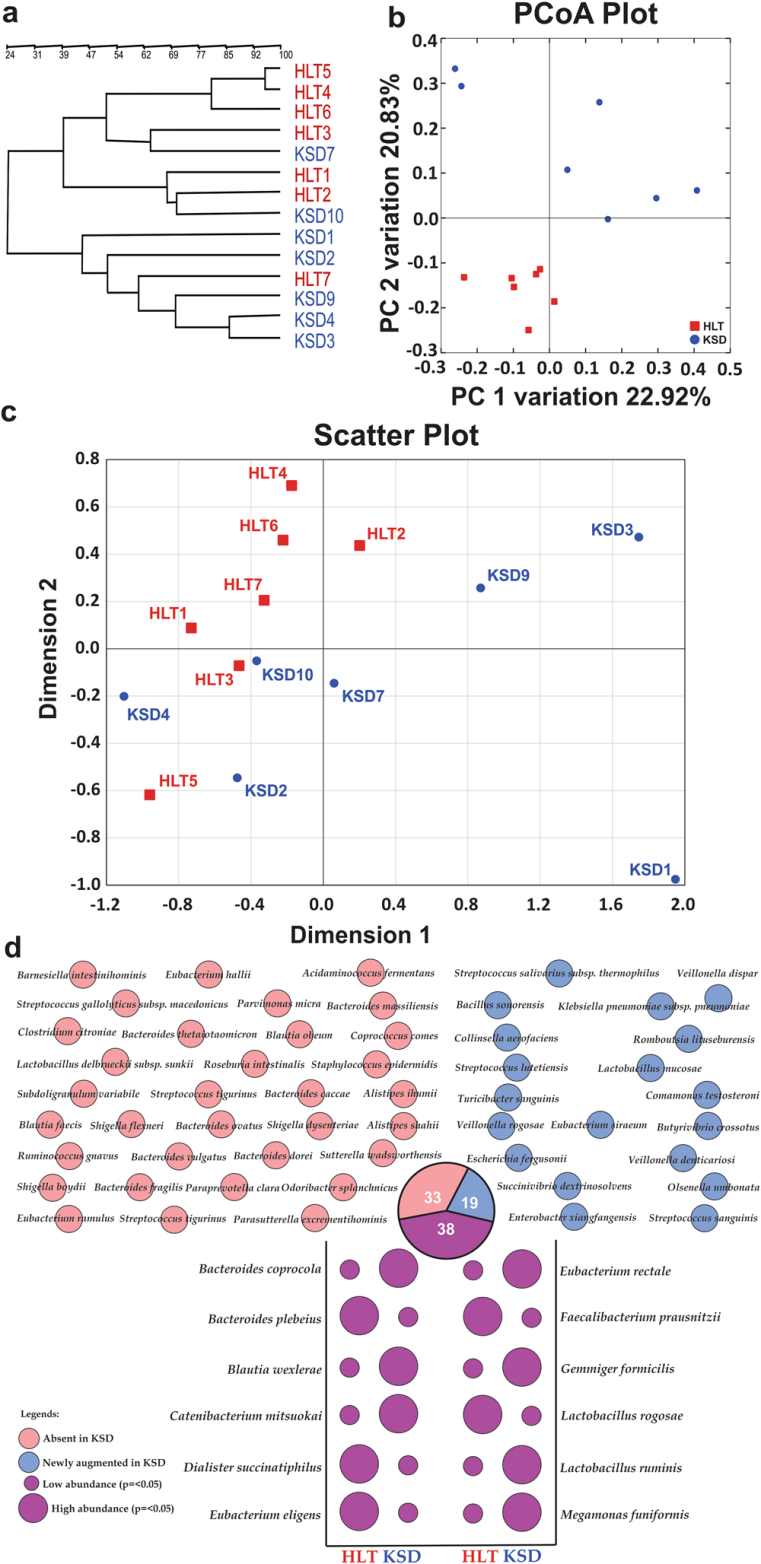
Compositional differences in eubacteria diversity in study subpopulations (HLT = 07 and KSD = 07). Clustering pattern of tested subpopulation through (**a**). PCR-DGGE fingerprint analysis in UPGMA algorithm, (**b**). PCoA plot and (**c**). IKF values on scattered plot derived from K-Shuff algorithm. (**d**) Species level diversity differences observed in HLT and KSD subjects, whereas shared species with significant difference (Welch’s test p < = 0.05) were annotated in bubble plot.﻿

**Figure 3:**
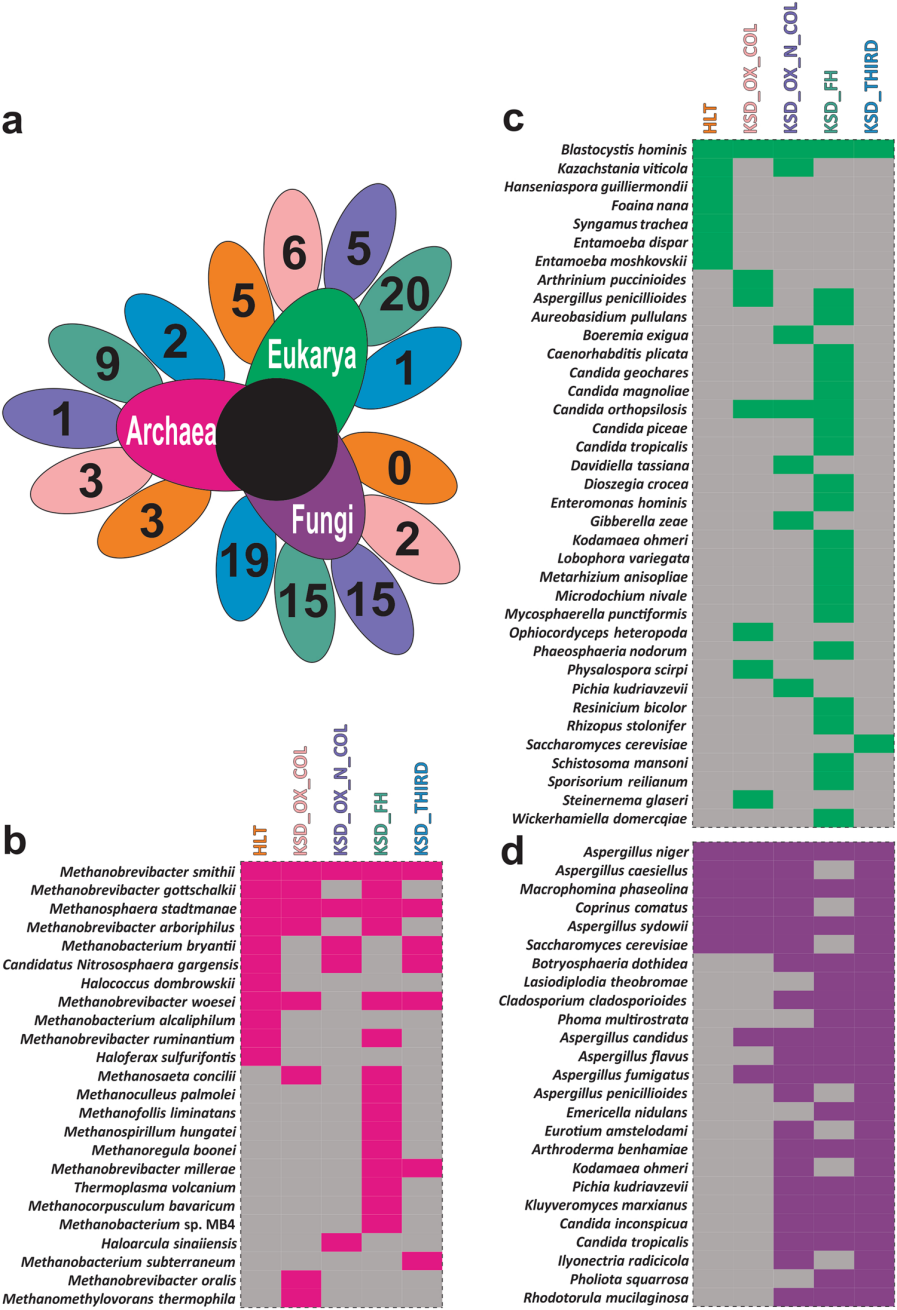
Trans-domain species diversity present in 5 groups (**a**) Diagrammatic illustration of unique Trans-domain species in each group, whereas comparison in HLT and KSD subjects only. The color of petal indicates the number of unique species either in HLT or KSD subject within 5 group. (**b**–**d**) Heatmap showing the species diversity in each five groups with pink (archaea), green (microeukaryotes) and violet (fungi) represents actual trans-domain components. Diversity was studied by the conserved genes such as 16S rRNA gene (archaea and eubacteria), 18S rRNA gene (microeukaryotes) and ITS region (fungi) sequencing. Presence and absence of gene bearing species data utilized for the representation.

