# Supplementary material for: Functional eubacteria species along with trans-domain gut inhabitants favour dysgenic diversity in oxalate stone disease
Source: Sci Rep. 2018 Nov 9;8:16598. doi: 10.1038/s41598-018-33773-5 (PMC6226508; doi:10.1038/s41598-018-33773-5)
Supplement: Supplementary file 1 — Supplementary Tables [file 41598_2018_33773_MOESM1_ESM.pdf]

Functional eubacteria species along with trans-domain gut inhabitants favour dysgenic diversity in oxalate stone disease

Mangesh Vasant Suryavanshi, Shrikant Subhash Bhute, Rahul Prakash Gune and Yogesh Shripad Shouche

Supplementary Table S1: Study subjects and their recorded metadata

Subject ID	Stone Episode	Stone Position	Family History	Diet	Age	Urine-Spp. Gravity	*Oxalobacter* Colonization
**‘Case-control study' recruited subjects**							
KSD1	Third	LUT	No	O/N	32	1.01	No
KSD2	Second	UUT	No	Veg	32	1.01	No
KSD3	Second	UUT	Yes	O/N	34	1	No
KSD4	Second	UUT	No	Veg	50	1.01	No
KSD7	Second	UUT	No	O/N	29	1.005	Yes
KSD9	Second	LUT	Yes	O/N	32	NaN	No
KSD10	Second	UUT	No	O/N	25	NaN	Yes
KSD12	Second	UUT	No	O/N	31	NaN	No
KSD13	Third	UUT	No	O/N	36	NaN	No
KSD14	Second	UUT	No	Veg	43	NaN	No
KSD20	Second	UUT	No	Veg	37	1.05	No
KSD21	Second	UUT	Yes	O/N	50	1.01	No
KSD22	Second	LUT	No	O/N	32	1.01	Yes
KSD23	Second	UUT	No	O/N	28	1.01	No
KSD24	Second	UUT	No	O/N	29	1.01	No
KSD25	Second	UUT	Yes	O/N	50	1.01	No
KSD26	Second	UUT	No	O/N	22	1.01	No
KSD27	Third	UUT	No	Veg	37	1.01	No
KSD28	Second	UUT	No	O/N	28	1.01	Yes
KSD29	Second	UUT	No	O/N	43	1.01	No
KSD30	Second	UUT	No	Veg	50	NaN	No

KSD31	Second	UUT	No	O/N	45	1.05	No
KSD32	Third	UUT	No	O/N	41	1	No
KSD33	Second	UUT	No	O/N	43	NaN	No
HLT1	No	NA	No	O/N	38	1.015	Yes
HLT2	No	NA	No	O/N	42	NaN	Yes
HLT3	No	NA	No	O/N	52	NaN	Yes
**Subject ID**	**Stone Episode**	**Stone Position**	**Family History**	**Diet**	**Age**	**Urine-Spp. Gravity**	***Oxalobacter* Colonization**
HLT4	No	NA	No	O/N	25	NaN	Yes
HLT5	No	NA	No	O/N	32	NaN	Yes
HLT6	No	NA	No	Veg	36	1.01	Yes
HLT7	No	NA	No	Veg	45	NaN	Yes
HLT8	No	NA	No	O/N	37	NaN	Yes
HLT9	No	NA	No	O/N	51	NaN	Yes
HLT10	No	NA	No	O/N	32	NaN	Yes
HLT11	No	NA	No	O/N	28	NaN	Yes
HLT12	No	NA	No	O/N	29	1	Yes
HLT13	No	NA	No	O/N	40	1.01	Yes
HLT14	No	NA	No	O/N	22	1.01	Yes
HLT15	No	NA	No	O/N	27	1.01	Yes
**‘Healthy-control study' recruited subjects (under HLT catogory)**							
CV1	No	NA	No	O/N	33	NaN	Yes
CV2	No	NA	No	O/N	34	NaN	Yes
CV3	No	NA	No	O/N	29	NaN	Yes
CV4	No	NA	No	O/N	37	NaN	Yes
CV5	No	NA	No	O/N	36	NaN	Yes
CV6	No	NA	No	O/N	34	NaN	Yes
CV7	No	NA	No	O/N	28	NaN	Yes
CV8	No	NA	No	O/N	39	NaN	Yes
CV9	No	NA	No	O/N	27	NaN	Yes
CV10	No	NA	No	O/N	28	NaN	Yes

CV11	No	NA	No	O/N	28	NaN	Yes
CV12	No	NA	No	O/N	30	NaN	Yes
CV13	No	NA	No	O/N	27	NaN	Yes
CV14	No	NA	No	Veg	25	NaN	Yes
CV15	No	NA	No	O/N	28	NaN	Yes
CV16	No	NA	No	O/N	28	NaN	Yes
**Subject ID**	**Stone Episode**	**Stone Position**	**Family History**	**Diet**	**Age**	**Urine-Spp. Gravity**	***Oxalobacter* Colonization**
CV17	No	NA	No	O/N	31	NaN	Yes
CV18	No	NA	No	O/N	28	NaN	Yes
CV19	No	NA	No	O/N	25	NaN	Yes
CV20	No	NA	No	O/N	25	NaN	Yes
CV21	No	NA	No	Veg	31	NaN	Yes
CV22	No	NA	No	O/N	30	NaN	Yes
CV23	No	NA	No	O/N	30	NaN	Yes
CV24	No	NA	No	O/N	27	NaN	Yes
CV25	No	NA	No	O/N	28	NaN	Yes
CV26	No	NA	No	O/N	40	NaN	Yes
CV27	No	NA	No	O/N	30	NaN	Yes
CV28	No	NA	No	O/N	28	NaN	Yes
CV29	No	NA	No	O/N	36	NaN	Yes
CV30	No	NA	No	O/N	30	NaN	Yes
CV31	No	NA	No	O/N	29	NaN	Yes
CV32	No	NA	No	O/N	27	NaN	Yes
CV33	No	NA	No	O/N	36	NaN	Yes
CV34	No	NA	No	O/N	38	NaN	Yes

LUT= Lower Urinary Tract position; UUT= Upper Urinary Tract position; NA= Not Any; O/N= Occasional Non-veg diet; NaN= None Recorded generated.

'Case-control study' recuited subjects are adopted from *Suryavanshi et. al. 2016*.

Supplementary Table S3: List of Eubacteria-functional species derived from targeted-metagenome

Functional species	KSD_HLT	KSD_OX_COL	KSD_OX_N_COL	KSD_FH	KSD_THIRD
***frc*-gene sequence taxonomy**					
**Ahrensia sp.**	7	0	0	0	0
**[Clostridium] difficile**	3	0	0	0	0
**Blautia hansenii**	3	0	0	0	0
**Prevotella buccae**	3	0	0	0	0
**Acinetobacter radioresistens**	2	0	0	0	0
**Eubacterium dolichum**	2	0	0	0	0
**Eubacterium ventriosum**	2	0	0	0	0
**[Ruminococcus] gnavus**	1	0	0	0	0
**Ancylobacter polymorphus**	1	0	0	0	0
**Clostridium tetani**	1	0	0	0	0
**Neisseria meningitidis**	1	0	0	0	0
**Oxalobacter formigenes**	2451	1164	0	0	0
**Yersinia pestis**	0	1587	0	69	0
**Salmonella enterica**	0	648	1	4	32
**uncultured bacterium**	0	377	0	0	0
**Yersinia pseudotuberculosis**	0	303	0	13	0
**Xanthobacter autotrophicus**	0	286	0	0	0
**Enterobacter cancerogenus**	0	54	0	0	0
**Serratia proteamaculans**	0	44	0	1	0
**Klebsiella pneumoniae**	0	37	11	0	34
**Enterobacter cloacae**	0	24	25	10	8
**Clostridium sp.**	0	24	9	8	26
**Shigella flexneri**	0	20	0	20	28
**Bacteroides vulgatus**	0	17	0	3	126
**Subdoligranulum variabile**	0	15	0	8	0
**Holdemania filiformis**	0	15	0	0	2
**Enterobacter sp.**	0	11	0	0	2
**Parabacteroides merdae**	0	7	1	0	14
**Eubacterium rectale**	0	6	0	2	42
**Parabacteroides distasonis**	0	6	0	0	60
**Arthrobacter aurescens**	0	6	0	0	0
**Edwardsiella tarda**	0	5	0	1	0
**Photorhabdus asymbiotica**	0	5	0	0	0
**Citrobacter koseri**	0	3	7	0	6
**Leptothrix cholodnii**	0	3	0	0	0

**Methylobacterium nodulans**	0	3	0	0	0
**Clostridium hylemonae**	0	2	0	2	2
**Mitsuokella multacida**	0	2	0	0	62
**Bacteroides ovatus**	0	2	0	0	8
**[Ruminococcus] obeum**	0	2	0	0	1
**Achromobacter piechaudii**	0	2	0	0	0
**Anaerostipes caccae**	0	2	0	0	0
**Prevotella marshii**	0	2	0	0	0
**Yersinia bercovieri**	0	2	0	0	0
**Yersinia mollaretii**	0	2	0	0	0
**Ethanoligenens harbinense**	0	1	0	1	2
**Bacillus sp.**	0	1	0	0	0
**Clostridium asparagiforme**	0	1	0	0	0
**Bifidobacterium adolescentis**	0	0	144	21	216
**Nocardiopsis dassonvillei**	0	0	57	6	0
**Bifidobacterium dentium**	0	0	15	0	100
**Bifidobacterium angulatum**	0	0	14	0	4
**Ruminococcus lactaris**	0	0	10	0	8
**Bifidobacterium catenulatum**	0	0	6	0	6
**Veillonella parvula**	0	0	5	0	0
**Yersinia enterocolitica**	0	0	5	0	0
**Lactobacillus ruminis**	0	0	4	0	13
**Clostridium saccharolyticum**	0	0	4	0	0
**Escherichia fergusonii**	0	0	4	0	0
**Edwardsiella ictaluri**	0	0	3	0	0
**Sutterella wadsworthensis**	0	0	2	0	5
**Acidovorax ebreus**	0	0	2	0	0
**Acidovorax sp.**	0	0	2	0	0
**Caldanaerobacter subterraneus**	0	0	2	0	0
**Citrobacter freundii**	0	0	2	0	0
**Lactobacillus salivarius**	0	0	2	0	0
**Prevotella bivia**	0	0	2	0	0
**Shigella sonnei**	0	0	1	4	8
**Lactobacillus plantarum**	0	0	1	0	1
**Bradyrhizobium genosp. TUXTLAS-23**	0	0	1	0	0
**Citrobacter rodentium**	0	0	1	0	0
**Cronobacter turicensis**	0	0	1	0	0

**Pantoea sp.**	0	0	2	0	0
**Serratia marcescens**	0	0	1	0	0
**Oligotropha carboxidovorans**	0	0	0	1064	0
**Actinobacillus pleuropneumoniae**	0	0	0	21	0
**Prevotella bergensis**	0	0	0	10	0
**Shigella boydii**	0	0	0	9	18
**Polaromonas sp. JS666**	0	0	0	5	1114
**Cryptobacterium curtum**	0	0	0	3	3
**Shigella sp.**	0	0	0	2	4
**Prevotella buccalis**	0	0	0	2	0
**Succinatimonas hippei**	0	0	0	1	1
**[Ruminococcus] torques**	0	0	0	1	0
**Clostridium kluyveri**	0	0	0	1	0
**Coprococcus catus**	0	0	0	1	0
**Flavobacteria bacterium BAL38**	0	0	0	1	0
**Prevotella nigrescens**	0	0	0	1	0
**Bacteroides coprophilus**	0	0	0	0	90
**Bacteroides stercoris**	0	0	0	0	41
**Bacteroides coprocola**	0	0	0	0	37
**Coprococcus comes**	0	0	0	0	12
**Bacteroides caccae**	0	0	0	0	10
**Prevotella ruminicola**	0	0	0	0	10
**Dorea longicatena**	0	0	0	0	8
**Eubacterium biforme**	0	0	0	0	8
**Acinetobacter baumannii**	0	0	0	0	6
**Bifidobacterium pseudocatenulatum**	0	0	0	0	4
**Eubacterium siraeum**	0	0	0	0	4
**Acinetobacter sp.**	0	0	0	0	3
**Porphyromonas gingivalis**	0	0	0	0	3
**Stenotrophomonas maltophilia**	0	0	0	0	3
**Bacteroides dorei**	0	0	0	0	2
**Bacteroides uniformis**	0	0	0	0	2
**Dialister invisus**	0	0	0	0	2
**Acinetobacter nosocomialis**	0	0	0	0	1
**Acinetobacter pittii**	0	0	0	0	1
**Bacteroides xylanisolvens**	0	0	0	0	1
**Bilophila wadsworthia**	0	0	0	0	1
**Eubacterium acidaminophilum**	0	0	0	0	1

**Eubacterium cellulosolvens**	0	0	0	0	1
**Megamonas hypermegale**	0	0	0	0	1
**Stenotrophomonas sp.**	0	0	0	0	1
***but*-gene sequence taxonomy**					
**[Clostridium] difficile**	0	1	0	12	0
**[Ruminococcus] gnavus**	0	3	0	0	0
**[Ruminococcus] obeum**	2	2	0	3	0
**[Ruminococcus] torques**	0	1	0	13	1
**Acidaminococcus fermentans**	10	14	0	0	18
**Actinomyces odontolyticus**	0	0	0	2	0
**Actinomyces sp.**	0	0	0	0	1
**Akkermansia muciniphila**	0	3	6	3	0
**Alistipes putredinis**	0	20	0	0	3
**Alistipes shahii**	1	7	0	0	1
**Alistipes sp.**	4	10	1	4	4
**Anaerostipes caccae**	6	12	0	0	0
**Arthrobacter aurescens**	0	396	3	18	0
**Arthrobacter sp.**	0	162	0	0	0
**Atopobium rimae**	0	2	0	0	1
**Azoarcus sp.**	0	4	0	0	0
**Bacillus cellulosilyticus**	2	0	0	2	0
**Bacteroides caccae**	0	1	2	2	2
**Bacteroides cellulosilyticus**	0	0	0	2	0
**Bacteroides coprocola**	0	24	0	24	8
**Bacteroides coprophilus**	0	0	0	6	68
**Bacteroides dorei**	6	16	6	8	20
**Bacteroides eggerthii**	7	16	2	8	10
**Bacteroides finegoldii**	0	0	0	0	2
**Bacteroides fragilis**	243	85	4	39	296
**Bacteroides helcogenes**	0	0	0	2	0
**Bacteroides intestinalis**	6	25	14	13	11
**Bacteroides ovatus**	1	7	3	5	11
**Bacteroides plebeius**	9	16	0	15	10
**Bacteroides sp.**	117	93	19	56	166
**Bacteroides stercoris**	12	16	4	8	12
**Bacteroides thetaiotaomicron**	0	2	0	20	2
**Bacteroides uniformis**	0	14	0	0	53
**Bacteroides vulgatus**	5	24	19	38	13
**Bacteroides xylanisolvens**	2	14	2	8	12
**Bifidobacterium**	103	85	537	1247	372

**adolescentis**					
**Bifidobacterium angulatum**	4	4	14	58	18
**Bifidobacterium animalis**	0	0	0	5	0
**Bifidobacterium bifidum**	4	8	12	39	46
**Bifidobacterium breve**	0	0	5	45	2
**Bifidobacterium catenulatum**	32	3	11	36	34
**Bifidobacterium dentium**	3	5	60	93	2
**Bifidobacterium longum**	108	173	974	2165	1924
**Bifidobacterium pseudocatenulatum**	23	0	0	45	60
**Bifidobacterium sp.**	4	12	42	126	157
**Bilophila wadsworthia**	0	0	1	1	1
**Blautia hansenii**	0	0	0	2	0
**Brachybacterium faecium**	0	1	0	0	0
**Brachyspira murdochii**	0	218	0	0	0
**butyrate-producing bacterium SS3/4**	5	2	0	0	6
**butyrate-producing bacterium SSC/2**	1	0	0	0	4
**Butyrivibrio fibrisolvens**	4	1	0	0	0
**Caldicellulosiruptor bescii**	0	6	0	0	0
**Caldicellulosiruptor saccharolyticus**	0	14	0	0	0
**Candidatus Solibacter usitatus**	0	0	1	0	0
**Candidatus Zinderia insecticola**	1	0	0	0	0
**Carboxydothermus hydrogenoformans**	6	0	0	0	0
**Citrobacter koseri**	0	9	7	0	6
**Citrobacter sp.**	0	4	1	0	0
**Citrobacter youngae**	0	2	0	0	0
**Clostridiales bacterium**	3	0	7	2	1
**Clostridium acetobutylicum**	0	0	0	0	2
**Clostridium asparagiforme**	0	8	0	10	0
**Clostridium beijerinckii**	0	0	0	0	2
**Clostridium bolteae**	0	0	0	3	7
**Clostridium botulinum**	0	538	0	22	2
**Clostridium carboxidivorans**	1	0	0	0	0
**Clostridium cellulolyticum**	0	0	0	0	1
**Clostridium hathewayi**	0	5	0	0	0

**Clostridium leptum**	0	2	0	0	1
**Clostridium ljungdahlii**	0	4	0	0	0
**Clostridium methylpentosum**	5	0	0	0	1
**Clostridium nexile**	2	0	0	4	2
**Clostridium phytofermentans**	0	0	0	11	0
**Clostridium scindens**	0	0	0	0	2
**Clostridium sp.**	29	11	3	12	15
**Clostridium thermocellum**	16	0	0	0	0
**Collinsella aerofaciens**	24	21	18	31	40
**Collinsella intestinalis**	0	0	0	2	3
**Coprobacillus sp.**	0	0	0	1	0
**Coprococcus catus**	2	1	2	1	0
**Coprococcus eutactus**	2	20	1	0	6
**Coprococcus sp.**	0	0	0	0	2
**Cronobacter sakazakii**	2	17	0	2	0
**Cronobacter turicensis**	0	0	0	1	0
**Cryptobacterium curtum**	0	0	3	3	0
**Desulfitobacterium hafniense**	0	0	1	0	0
**Dialister invisus**	0	0	0	32	0
**Dialister micraerophilus**	0	3	0	1	0
**Dickeya dadantii**	0	1	0	0	0
**Dinoroseobacter shibae**	0	4	0	0	0
**Dorea formicigenerans**	0	2	0	0	0
**Dorea longicatena**	6	0	3	0	15
**Edwardsiella tarda**	0	1	0	0	0
**Eggerthella lenta**	0	0	0	0	8
**Eggerthella sp.**	0	0	0	0	2
**Enterobacter aerogenes**	1	0	0	0	1
**Enterobacter cancerogenus**	0	4	0	0	0
**Enterobacter cloacae**	3	200	51	19	6
**Enterobacter sp.**	0	71	12	10	0
**Erysipelotrichaceae bacterium 5_2_54FAA**	2	0	1	0	3
**Escherichia coli**	1173	26102	4440	3210	802
**Escherichia fergusonii**	4	19	4	4	4
**Escherichia sp.**	12	256	33	28	6
**Eubacterium biforme**	0	0	0	2	0
**Eubacterium cellulosolvens**	1	1	0	0	0
**Eubacterium dolichum**	0	4	0	0	2
**Eubacterium eligens**	0	1	0	0	0
**Eubacterium hallii**	0	53	11	11	3

**Eubacterium limosum**	0	0	0	66	0
**Eubacterium rectale**	783	1240	282	381	198
**Eubacterium siraeum**	0	0	0	0	3
**Eubacterium ventriosum**	0	4	0	0	4
**Faecalibacterium prausnitzii**	270	402	243	194	176
**Fibrobacter succinogenes**	0	0	0	5	0
**Fusobacterium ulcerans**	2	0	0	0	0
**Gardnerella vaginalis**	0	0	0	4	0
**Haemophilus haemoglobinophilus**	0	0	0	1	0
**Haemophilus influenzae**	3	0	0	0	0
**Holdemania filiformis**	12	0	0	23	2
**Ilyobacter polytropus**	1	0	0	0	0
**Klebsiella pneumoniae**	14	393	13	39	126
**Klebsiella sp.**	0	17	0	0	4
**Klebsiella variicola**	1	39	0	8	12
**Kocuria rhizophila**	0	1	0	18	0
**Lactobacillus delbrueckii**	0	4	0	0	0
**Lactobacillus gasseri**	7	0	0	0	0
**Lactobacillus johnsonii**	0	0	0	0	3
**Lactobacillus plantarum**	1	0	0	0	0
**Lactobacillus ruminis**	0	6	18	42	9
**Lactococcus lactis**	0	0	0	1	0
**Legionella pneumophila**	1	0	0	0	0
**Lutiella nitroferrum**	0	2	0	0	0
**Megamonas hypermegale**	1	0	0	1	0
**Megasphaera micronuciformis**	0	1	0	0	0
**Methylococcus capsulatus**	0	0	0	0	4
**Mitsuokella multacida**	0	18	0	5	30
**Neisseria flavescens**	1	0	0	0	0
**Neisseria meningitidis**	4	3	0	0	0
**Nitrosomonas europaea**	0	4	0	0	0
**Olsenella uli**	0	0	0	1	0
**Paenibacillus sp.**	2	0	0	0	0
**Paludibacter propionicigenes**	0	0	0	0	1
**Pantoea sp.**	0	4	0	2	0
**Pantoea vagans**	0	1	0	0	0
**Parabacteroides distasonis**	53	16	5	13	44
**Parabacteroides johnsonii**	8	23	8	8	24
**Parabacteroides merdae**	32	57	38	44	38

**Parabacteroides sp.**	2	0	0	0	1
**Pectobacterium atrosepticum**	0	2	0	0	0
**Pectobacterium carotovorum**	0	7	0	0	0
**Pectobacterium wasabiae**	0	3	0	0	0
**Pediococcus acidilactici**	4	0	0	0	0
**Pelobacter carbinolicus**	0	0	0	6	0
**Peptoniphilus sp. oral taxon 836**	0	1	0	0	0
**Peptostreptococcus anaerobius**	2	0	0	0	0
**Photorhabdus asymbiotica**	1	0	0	0	0
**Photorhabdus luminescens**	3	0	0	0	0
**Planococcus donghaensis**	1	0	0	0	0
**Porphyromonas gingivalis**	0	1	0	0	0
**Prevotella bivia**	0	0	4	0	0
**Prevotella buccae**	2	0	1	0	0
**Prevotella copri**	322	216	206	42	77
**Prevotella loescheii**	0	0	6	0	0
**Prevotella marshii**	4	0	0	0	0
**Prevotella melaninogenica**	0	2	0	0	0
**Prevotella oris**	0	0	0	0	2
**Prevotella salivae**	5	9	1	4	4
**Prevotella sp.**	12	0	6	0	0
**Prevotella veroralis**	10	2	0	0	0
**Propionibacterium freudenreichii**	0	0	1	0	0
**Pseudoflavonifractor capillosus**	8	0	0	0	15
**Pseudomonas aeruginosa**	0	21	0	0	0
**Rahnella sp.**	0	1	0	0	0
**Renibacterium salmoninarum**	0	2	0	0	0
**Rhodobacter sphaeroides**	0	0	0	0	5
**Rhodopseudomonas palustris**	0	0	0	0	3
**Robiginitalea biformata**	3	0	0	0	0
**Roseburia intestinalis**	13	22	4	0	2
**Roseburia inulinivorans**	275	167	55	77	50
**Roseburia sp.**	1	0	0	0	0
**Rothia dentocariosa**	0	0	0	8	0
**Rothia mucilaginosa**	0	0	0	18	0

**Ruminococcus albus**	0	12	7	4	2
**Ruminococcus bromii**	0	4	0	0	2
**Ruminococcus champanellensis**	3	0	0	1	3
**Ruminococcus lactaris**	1	6	0	9	0
**Ruminococcus sp.**	13	6	8	66	12
**Salinispora tropica**	0	3	0	0	0
**Salmonella bongori**	0	0	0	1	0
**Salmonella enterica**	71	512	24	226	1
**Serratia marcescens**	0	3	0	0	0
**Serratia odorifera**	0	5	0	0	0
**Serratia proteamaculans**	3	4	0	0	0
**Serratia symbiotica**	0	1	0	0	0
**Shigella boydii**	23	295	53	35	6
**Shigella dysenteriae**	4	166	4	14	6
**Shigella flexneri**	75	856	190	123	136
**Shigella sonnei**	4	128	7	9	6
**Shigella sp.**	16	246	40	34	4
**Sodalis glossinidius**	0	0	6	0	0
**Streptococcus parasanguinis**	0	0	0	5	0
**Streptococcus pyogenes**	0	0	4	0	0
**Streptococcus salivarius**	2	0	0	0	0
**Succinatimonas hippei**	0	1	0	0	0
**Sutterella wadsworthensis**	6	0	2	6	5
**Syntrophothermus lipocalidus**	16	0	0	0	0
**Thermoanaerobacter brockii**	0	4	0	6	4
**Thermoanaerobacter ethanolicus**	0	0	0	6	4
**Thermoanaerobacter pseudethanolicus**	0	0	2	9	6
**Thermoanaerobacter sp.**	0	0	2	24	16
**Thermoanaerobacterium thermosaccharolyticum**	1	0	0	0	0
**Veillonella atypica**	0	0	0	0	2
**Veillonella parvula**	0	58	1	0	6
**Veillonella sp.**	0	0	0	1	0
**Yersinia frederiksenii**	0	13	0	0	0
**Yersinia pestis**	0	0	0	38	0
***buk*-gene sequence taxonomy**					
**[Clostridium] bartlettii**	0	2	22	16	0
**[Ruminococcus] torques**	0	0	0	0	1
**Abiotrophia defectiva**	0	0	2	0	0
**Alistipes sp.**	0	1	0	0	0

**Bacteroides caccae**	0	0	22	6	18
**Bacteroides coprocola**	0	3	0	0	0
**Bacteroides coprophilus**	0	0	0	0	1
**Bacteroides dorei**	0	2	0	0	0
**Bacteroides eggerthii**	0	2	0	0	0
**Bacteroides fragilis**	687	174	558	996	475
**Bacteroides helcogenes**	0	0	3	4	0
**Bacteroides intestinalis**	0	3	0	0	0
**Bacteroides ovatus**	58	118	420	434	72
**Bacteroides plebeius**	0	0	0	0	2
**Bacteroides sp.**	837	229	1298	1836	579
**Bacteroides stercoris**	0	2	1	3	0
**Bacteroides thetaiotaomicron**	513	0	541	652	693
**Bacteroides vulgatus**	0	0	25	40	0
**Bacteroides xylanisolvens**	4	2	0	8	4
**Bifidobacterium adolescentis**	0	0	0	0	5
**Bifidobacterium angulatum**	2	0	0	0	0
**butyrate-producing bacterium L2-50**	76	115	70	128	399
**butyrate-producing bacterium SSC/2**	1	0	0	0	0
**Catenibacterium mitsuokai**	0	0	4	0	0
**Clostridiales bacterium**	0	7	23	0	0
**Clostridium botulinum**	0	0	0	0	2
**Clostridium nexile**	0	0	0	0	2
**Clostridium phytofermentans**	0	0	0	3	0
**Clostridium sp.**	263	244	170	322	798
**Clostridium tetani**	0	0	32	0	0
**Coprococcus comes**	0	0	81	0	492
**Coprococcus eutactus**	108	0	0	0	1070
**Eubacterium biforme**	0	0	0	26	0
**Eubacterium hallii**	0	0	38	96	122
**Eubacterium rectale**	327	157	209	360	294
**Eubacterium ventriosum**	90	4	6	0	0
**Faecalibacterium prausnitzii**	0	0	0	0	1
**Klebsiella pneumoniae**	0	0	0	0	9
**Lachnospiraceae bacterium**	1	0	0	0	0
**Parabacteroides johnsonii**	0	2	0	0	0
**Parabacteroides merdae**	0	2	0	0	2
**Prevotella buccalis**	0	0	0	2	0

**Prevotella copri**	9	0	0	0	3
**Roseburia inulinivorans**	0	2	0	0	0
**Ruminococcus sp.**	0	0	0	0	2
**Saccharomyces cerevisiae**	0	0	0	0	4
**Shuttleworthia satelles**	0	2	0	0	0
**Streptococcus sanguinis**	3	0	0	0	0

Supplementary Table S4: 16S rRNA gene clone library analysis summary

Subject ID	Sequences (N)	dOTU	Shannon (H)	Simpson (D)	IKF	sOTU	K/As	N/As
HLT1	376	56	2.31906	0.247688	0.274	121	307	69
HLT2	334	71	3.23447	0.0820701	0.3534	139	227	107
HLT3	381	63	2.5302	0.233112	0.2853	120	237	144
HLT4	332	55	2.60901	0.139228	0.3156	120	249	83
HLT5	342	36	1.59354	0.423111	0.2237	77	272	70
HLT6	387	68	3.06645	0.104203	0.3303	147	258	129
HLT7	350	75	2.84163	0.166811	0.3061	143	271	79
KSD1	342	82	3.44493	0.0748229	0.2467	129	125	217
KSD2	336	20	1.33636	0.420291	0.2578	42	317	19
KSD3	349	28	2.11976	0.19308	0.2974	53	334	15
KSD4	377	20	1.62897	0.367007	2374	45	247	130
KSD7	349	48	2.63289	0.167161	0.3137	78	231	118
KSD9	469	42	2.47629	0.135404	0.3449	107	412	57
KSD10	333	43	2.37667	0.221408	0.2959	87	197	136

dOTU-Number of OTUs formed at 97 % sequence similarity using DOTUR algorithm.

IKF-Index derived from K Shuff algorithm (Jangid et. al., 2016).

sOTU-Number of OTUs formed at 97 % sequence similarity using Silva123 database by open-reference OTU generation in QIIME.

K/As or N/As-Number of library sequences gained or fails respectively to taxonomic assignments at 97 % sequence similarity using EzTaxon database to the sOTUs.

Supplementary Table S5: List of Eubacterial species (≥97% Identity cut-off) derived from 16S rRNA gene clone library

OTU ID	Similarity (%)	EzTaxon_Taxonomy
338145	99.21	Olsenella umbonata
184745	100	Collinsella aerofaciens
180133	99.13	Barnesiella intestinihominis
772282	99.69	Alistipes shahii
216599	99.53	Alistipes ihumii
199716	99.89	Bacteroides dorei
174646	98.91	Bacteroides thetaiotaomicron
4454586	99.37	Odoribacter splanchnicus
327050	99.84	Bacteroides massiliensis
165118	99.2	Paraprevotella clara
332317	99.52	Bacteroides ovatus
183603	99.69	Bacteroides fragilis
184610	99.67	Bacteroides caccae
184753	99.67	Bacteroides vulgatus
213813	100	Bacteroides plebeius
359538	99.77	Bacteroides stercoris
209685	99.78	Bacteroides coprocola
350277	100	Bacteroides uniformis
denovo303	99.78	Prevotella stercorea
174842	98.13	Parabacteroides distasonis
307571	99.06	Prevotella copri
denovo296	99.89	Parabacteroides merdae
3385021	100	Staphylococcus epidermidis
4369988	99.85	Streptococcus gallolyticus subsp. macedonicus
586141	100	Lactobacillus delbrueckii subsp. sunkii
4320317	99.84	Streptococcus tigurinus
131660	99.68	Lactobacillus delbrueckii subsp. indicus
2901965	99.9	Streptococcus vestibularis
802262	99.89	Streptococcus sanguinis
579608	99.87	Streptococcus salivarius subsp. thermophilus
338757	99.88	Lactobacillus mucosae
4447476	99.09	Bacillus sonorensis
3244896	99.76	Streptococcus infantarius subsp. coli
178213	99.7	Lactobacillus ruminis
289925	99.44	Streptococcus lutetiensis
denovo390	99.22	Turicibacter sanguinis
1978955	99.24	Subdoligranulum variabile
2101287	99.68	Eubacterium ramulus
185603	98.88	Coprococcus comes
197675	99.77	Eubacterium hallii

OTU ID	Similarity (%)	EzTaxon_Taxonomy
3376513	99.06	Ruminococcus gnavus
105440	99.73	Blautia faecis
4481427	99.7	Roseburia intestinalis
2582660	99.85	Blautia obeum
denovo516	98.17	Acidaminococcus fermentans
851704	98.4	Parvimonas micra
4381303	99.08	Clostridium citroniae
16054	99.61	Ruminococcus callidus
187932	99.36	Blautia luti
181016	100	Eubacterium siraeum
13811	99.05	Mitsuokella multacida
174654	99.88	Ruminococcus bromii
514988	97.5	Romboutsia lituseburensis
270382	98.84	Romboutsia ilealis
4475758	99.15	Veillonella dispar
denovo273	99.56	Dialister succinatiphilus
2313540	100	Lactobacillus rogosae
181193	99.89	Butyrivibrio crossotus
193975	99.88	Coprococcus catus
190679	99	Coprococcus eutactus
4422456	98.79	Veillonella rogosae
4374302	100	Dorea formicigenerans
3754926	99.89	Anaerostipes hadrus
4391625	99.21	Veillonella denticariosi
181543	99.76	Dorea longicatena
2229500	99.84	Gemmiger formicilis
223773	98.78	Megamonas funiformis
149335	99.26	Mitsuokella jalaludinii
191797	98.77	Clostridium celatum
4358919	100	Roseburia inulinivorans
264967	99.36	Megasphaera indica
176269	100	Eubacterium eligens
195207	99.78	Roseburia faecis
266210	99.68	Megasphaera elsdenii
197004	100	Eubacterium rectale
4478125	99.68	Faecalibacterium prausnitzii
193623	100	Holdemanella biformis
4480861	97.81	Catenibacterium mitsuokai
denovo503	99.89	Blautia wexlerae
2201995	99.68	Sutterella wadsworthensis
112891	99.11	Parasutterella excrementihominis
509447	100	Comamonas testosteroni
4344722	99.22	Shigella boydii

OTU ID	Similarity (%)	EzTaxon_Taxonomy
566243	99.69	Shigella dysenteriae
4457268	99.68	Shigella flexneri
231787	99.49	Escherichia fergusonii
4333020	99.77	Enterobacter cloacae subsp. cloacae
2529285	99.77	Enterobacter xiangfangensis
197286	99.67	Klebsiella pneumoniae subsp. pneumoniae
524117	97.2	Succinivibrio dextrinosolvens

Supplementary Table S6: List of Trans-domain species derived from targeted-metagenome

**Trans-domain species**	**KSD_HLT**	**KSD_OX_COL**	**KSD_OX_N_COL**	**KSD_FH**	**KSD_THIRD**
**18S rRNA gene sequence taxonomy**					
Blastocystis hominis	4188	15	1	112	860
Kazachstania viticola	371	0	1381	0	0
Hanseniaspora guilliermondii	6	0	0	0	0
Foaina nana	3	0	0	0	0
Syngamus trachea	2	0	0	0	0
Entamoeba dispar	1	0	0	0	0
Entamoeba moshkovskii	1	0	0	0	0
Arthrinium puccinioides	0	1	0	0	0
Aspergillus penicillioides	0	1	0	3	0
Aureobasidium pullulans	0	0	0	6	0
Boeremia exigua	0	0	1	0	0
Caenorhabditis plicata	0	0	0	1	0
Candida geochares	0	0	0	3748	0
Candida magnoliae	0	0	0	1	0
Candida orthopsilosis	0	2	1	3	0
Candida piceae	0	0	0	1	0
Candida tropicalis	0	0	0	14	0
Davidiella tassiana	0	0	1	0	0
Dioszegia crocea	0	0	0	1	0
Enteromonas hominis	0	0	0	7	0
Gibberella zeae	0	0	1	0	0
Kodamaea ohmeri	0	0	0	1	0
Lobophora variegata	0	0	0	1	0
Metarhizium anisopliae	0	0	0	1	0

**Trans-domain species**	**KSD_HLT**	**KSD_OX_COL**	**KSD_OX_N_COL**	**KSD_FH**	**KSD_THIRD**
Microdochium nivale	0	0	0	1	0
Mycosphaerella punctiformis	0	0	0	1	0
Ophiocordyceps heteropoda	0	1	0	0	0
Phaeosphaeria nodorum	0	0	0	1	0
Physalospora scirpi	0	1	0	0	0
Pichia kudriavzevii	0	0	739	0	0
Resinicium bicolor	0	0	0	1	0
Rhizopus stolonifer	0	0	0	1	0
Saccharomyces cerevisiae	0	0	0	0	4
Schistosoma mansoni	0	0	0	8	0
Sporisorium reilianum	0	0	0	7	0
Steinernema glaseri	0	1	0	0	0
Wickerhamiella domercqiae	0	0	0	1	0
**ITS region sequence taxonomy**					
Aspergillus niger	71	71	96	74	29
Aspergillus caesiellus	42	188	1	0	1
Macrophomina phaseolina	41	5	142	144	16
Coprinus comatus	5	5	2	0	8
Aspergillus sydowii	3	75	0	4	1
Saccharomyces cerevisiae	2	147	5	0	7
Botryosphaeria dothidea	0	0	136	3	1
Lasiodiplodia theobromae	0	0	0	4	1
Cladosporium cladosporioides	0	0	35	4	18

Trans-domain species	KSD_HLT	KSD_OX_COL	KSD_OX_N_COL	KSD_FH	KSD_THIRD
Phoma multirostrata	0	0	0	2	1
Aspergillus candidus	0	1	6	14	4
Aspergillus flavus	0	0	1	5	17
Aspergillus fumigatus	0	1	42	2	2
Aspergillus penicillioides	0	0	4	0	39
Emericella nidulans	0	0	0	22	1
Eurotium amstelodami	0	0	55	0	14
Arthroderma benhamiae	0	0	3	47	1
Kodamaea ohmeri	0	0	88	0	7
Pichia kudriavzevii	0	0	68	98	13
Kluyveromyces marxianus	0	0	4	6	1
Candida inconspicua	0	0	74	6	1
Candida tropicalis	0	0	97	8	6
Ilyonectria radicicola	0	0	24	0	2
Pholiota squarrosa	0	0	0	3	1
Rhodotorula mucilaginosa	0	0	5	1	1
**Archaeal 16S rRNA gene sequence taxonomy**					
Methanobrevibacter smithii	4028	22452	3418	10206	9126
Methanobrevibacter gottschalkii	2507	1	0	2	0
Methanosphaera stadtmanae	1333	3850	310	1306	3071
Methanobrevibacter arboriphilus	360	1	0	1	0
Methanobacterium bryantii	98	0	1	0	1
Candidatus Nitrososphaera gargensis	49	0	1	0	1

Trans-domain species	KSD_HLT	KSD_OX_COL	KSD_OX_N_COL	KSD_FH	KSD_THIRD
Halococcus dombrowskii	7	0	0	0	0
Methanobrevibacter woesei	4	3	0	17	2
Methanobacterium alcaliphilum	4	0	0	0	0
Methanobrevibacter ruminantium	3	0	0	8	0
Haloferax sulfurifontis	2	0	0	0	0
Methanosaeta concilii	0	2	0	288	0
Methanoculleus palmolei	0	0	0	41	0
Methanofollis liminatans	0	0	0	19	0
Methanospirillum hungatei	0	0	0	15	0
Methanoregula boonei	0	0	0	9	0
Methanobrevibacter millerae	0	0	0	9	1
Thermoplasma volcanium	0	0	0	9	0
Methanocorpusculum bavaricum	0	0	0	5	0
Methanobacterium sp. MB4	0	0	0	1	0
Haloarcula sinaiiensis	0	0	1	0	0
Methanobacterium subterraneum	0	0	0	0	6
Methanobrevibacter oralis	0	2	0	0	0
Methanomethylovorans thermophila	0	5	0	0	0
