# Supplementary material for: Functional eubacteria species along with trans-domain gut inhabitants favour dysgenic diversity in oxalate stone disease
Source: Sci Rep. 2018 Nov 9;8:16598. doi: 10.1038/s41598-018-33773-5 (PMC6226508; doi:10.1038/s41598-018-33773-5)
Supplement: Supplementary file 3 — Supplementary File S7 [file 41598_2018_33773_MOESM3_ESM.doc]

**Functional eubacteria species along with trans-domain gut inhabitants favour dysgenic diversity in oxalate stone disease**

Mangesh Vasant Suryavanshi, Shrikant Subhash Bhute, Rahul Prakash Gune and Yogesh Shripad Shouche

# Supplementary File S7: Materials and methods used for data generation

**Sample collection and Processing**

We collected the fecal sample, 24h urine and surgically removed kidney stone samples from KSD, and fecal sample and 24h urine from HLT subjects. Only the fecal samples were used for bacterial diversity and targeted metagenomic analysis for this study. The qPCR primers used for this study were the same as described earlier ^1^ and in addition for the *Lactobacillus plantarum* species quantification Lplant_F 5’-TTACATTTGAGTGAGTGGCGAACT-3’ and Lplant_R 5’-AGGTGTTATCCCCCGCTTCT-3’ primers were used ^2^.

Thus, the subjects were grouped into five categories with four subjects in each group viz. KSD with family history (KSD_FH), KSD O. *formigenes* colonizer (KSD_OX_COL), KSD O. *formigenes* non-colonizer (KSD_OX_N_COL), KSD with third episode (KSD_THIRD) and Healthy control (HLT). Total community DNA was extracted from each faecal samples using QIAmp DNA Stool Mini kit (Qiagen, Madison USA) as per manufacturer’s protocol. The concentration of resulting DNA was measured using Nanodrop-1000, (Thermo Scientific, USA). DNA concentration was normalised to 100 ng/µl and used as template for amplification bacterial genes.

**Trans-domain diversity analysis**

For molecular detection of kind of diversity present into the study for comparison, we targeted groups of gut inhabitants for its diversity analysis through different gene amplicon sequencing.

For the trans-domain diversity, we used 18S rRNA gene for Eukaryotes, 16S rRNA gene for Archaebacteria and ITS gene for fungi to access across the study samples. These approaches and number of genes targeted for study are represented in **Supplementary File S7-Table 1**. For accessing the diversity from each target gene, we performed amplicon library preparation and sequence generation through ion torrent platform. For accessing the eubacteria species level diversity, we employed clone library preparation and Sanger sequencing methods.

**Amplicon gene sequencing on Ion Torrent and bioinformatics analysis**

All the described genes were amplified using AmpliTaq Gold PCR Master Mix (Life Technologies, USA). The archeal 16S, eukaryotic 18S and fungal ITS1 genes were PCR amplified using primers listed in Supplementary table 1. The resulting PCR products were purified using Agencourt AMPure XP DNA purification Bead (Beckman Coulter, USA) and quantified using Nanodrop-1000 (Thermo Scientific, USA). Then, PCR products of each gene and each sample of five groups were pooled by mixing equal quantities of concentration normalized PCR products. This way we obtained five pools for archaeal 16S rRNA, eukaryotic 18S rRNA, fungal ITS, *frc-, but-* and *buk-*gene seperately. All the pooled samples were then sequenced as described earlier. Since, fungal ITS, *but-* and *buk-*gene amplicons varied in length, we fragmented 100 ng of it with Ion Shear Enzyme mix (Ion Xpress Plus Fragment Library preparation kit, Life Technologies) for 20 min and 200 bp size fragments were selected before adapter ligation step.

Resulting PCR products were end repaired and ligated with sample specific barcode adaptor as explained in Ion Xpress™ Plus gDNA Fragment Library Preparation user guide. Prior to sequencing, fragment size distribution and molar concentrations of amplicons were assessed on Bioanalyser 2100 (Agilent Technologies, USA) using High Sensitivity DNA Analysis Kit. All amplicons were diluted to the lowest molar concentration and pooled into sets of 10 samples. Emulsion PCR was carried out on Ion OneTouchTM System using Ion OneTouch™ 200 Template Kit v2 DL (Life Technologies) as explained in Ion OneTouchTM 200 Template Kit v2 user manual. The resulting template positive Ion Sphere particles were enriched using Ion OneTouch ES system and sequencing of amplicon libraries was carried out on 316 chips using Ion Torrent PGM system and Ion Sequencing 200 kit following the user guide: Ion PGM™ Sequencing 200 Kit v2.

The fastq sequence files for the various amplicon genes were uploaded and analyzed using the MetaGenome Rapid Annotation with Subsystem Technology (MG-RAST) server, version 3.6 53. The raw reads underwent the quality filtering steps and host specific reads were excluded. Phylogenetic analysis was performed using default setting as detailed in MG-RAST manual version 3.6 revision 3 (ftp://ftp.metagenomics.anl.gov/data/manual/mg-rast-manual.pdf). Organism abundance up to species level was obtained by using phylogenetic analysis with Best Hit Classification approach search with minimum e-value and identity of 1e-5 and 90% respectively.

**DGGE analysis of the community DNA**

DGGE fingerprinting strategy was used to obtain community structure using 16S rRNA gene specific primers for accessing total eubacterial diversity of randomly selected subpopulation. 16S rRNA gene was utilized as molecular marker using modified linker primers GC-341F (5′-CGCCCGCCGCGCGCGGCGGGCGGGGCGGGGGCACGGGGGGCCTACGGGAGGCAGCAG-3′) and 517R (5′-ATTACCGCGGCTGCTGG-3′) as described earlier (Bano & Hollibaugh 2002). The PCR products were subjected to DGGE in 10% acrylamide: bis acrylamide (37.5:1) gel with a gradient of 40% to 60%. The electrophoresis was performed using DCode Universal Mutation Detection System (BioRad, Hercules, CA, USA) in 1 X TAE buffer (40 mM Tris, 20 mM Sodium acetate, 1 mM EDTA) at 80 V and 60 ºC for 18 h. The gel was stained with SYBR Gold (Invitrogen) for 20 minutes, visualized using SynGene G: box gel documentation system and analyzed using GeneTools software packages (SynGene, Cambridge, UK). Pair-wise band matching based community similarities were carried out using the Dice index and dendrograms were constructed using UPGMA methods.

**Species level diversity analysis for eubacteria**

From subpopulation, 16S rRNA gene was amplified using universal primers for bacteria as Eub8F and Eub907R ^3^. The PCR products were purified by gel elution using Gene Elute Gel Extraction Kit (Sigma-Aldrich, St Louis USA), ligated into pCR4® TOPO vector supplied with the TOPO TA cloning kit (Invitrogen, San Diego, USA) and clone generated by following the manufacturer’s instructions. Sequence generation and processing of raw reads to non-ambiguous, chimeric artefacts-free 16S rRNA gene sequences from each sample was done as described previously ^4^. These sequences were then pooled into single FASTA file and analyzed using QIIME software (Quantitative Insight into Microbial Ecology) version 1.8.0 ^5^. Briefly, UCLUST was used to cluster sequences to obtain OTUs at 97% similarity; representative sequence from each OTU was selected, aligned using PyNAST and OTU sequences were assigned to taxonomy using RDP classifier. Finally, OTU table was generated by tabulating the number of times an OTU was found in each sample with its taxonomic identification based on Greengene 13.8 database release, this OTU table was used for subsequent analysis. Alpha diversity indices and rarefaction analysis was performed within QIIME environment. Additionally, beta diversity analysis was performed using unweighted Unifrac and Principle Coordinate Analysis (PCoA) was carried out.

Taxonomic identification for the generated sequences picked from the cultured bacterial 16S rRNA gene database. We utilized the OTU table derived from open reference OTU picking method against Greengene 13.8 database release for the generation of species level diversity table. Representative sequence from each OTU was blast analysed in the EzTaxon-e server at ‘<https://www.ezbiocloud.net/identify>’ (database: 2017.05.11) and first blast hit with taxonomy and percentage of identity was recorded. Depending upon the percentage identity value we assigned the taxonomic rank to each of these representative sequences and ultimately to each OTUs from an OTU table. These added taxonomic attributes were used for the further analyses.

**Imputed metagenomic functional property of bacterial community**

For inferring the metabolic capabilities of bacterial community, we utilized a computational approach: PICRUSt tool (phylogenetic investigation of communities by reconstruction of unobserved states) ^6^. PICRUSt analysis reference based OTU picking was performed within QIIME environment and resulting biom file was imported to PICRUSt online tool at http://huttenhower.sph.harvard.edu/galaxy/. In this online tool; functional predictions were made using KEGG Orthology (KO) database, using default settings. Further, the statistical analysis of predicted metagenome was done using STAMP - version 2.0.2 ^7^.

**Statistics and data representation**

For statistical applications, we used GraphPad prism v6.01 software (GraphPad Software, La Jolla California USA). Hypergeometric test functions and shared phylotype network analysis were done in R software ^8^. For quantitation of genes Man-Whitney U test was used. For biomarker and eubacterial family identification, we employed the Welch’s t-test and Kruskal-Wallis test with Bonferroni correction for with FDR screenings. For imputed metagenome and species level eubacterial diversity analysis in HLT and KSD groups we applied non-parametric t-test for the significance value determination.

**Supplementary File S7-Table 1: Primers used during the PCR amplification of functional, archaeal, eukaryotic and fungal communities.**

| **Domain/Group** | **Primer Name** | **Sequence (5’-3’)** | **Product (bp)** | **Reference** |
| --- | --- | --- | --- | --- |
| Eubacteria  (16S rRNA gene) | Eub8F | AGAGTTTGATCCTGGCTCAG | 901 | 3 |
|  | Eub907R | CCGTCAATTCCTTTRAGTTT |  |  |
| OMBS  (*frc-*gene) | frc171-F | CTSTAYTTCACSATGCTSAAC | 135 | 9 |
|  | frc306-R | GDSAAGCCCATVCGRTC |  |  |
| Butyrate producer  (*but- and buk-*gene) | But-2F | GGWATWGGMGSYATGCC | Varied | 10 |
|  | But-2R | AARTCAASCTGKCCDC |  |  |
|  | Buk-2F | ATWAATCCWGGTTCWACWTCWACMAA | Varied |  |
|  | Buk-2R | TGCYTTYTGGTTGAGYGC |  |  |
| Archaea  (16S rRNA gene) | Archea806F | ATTAGATACCCSBGTAGTCC | 194 | 11 |
|  | Archea1000R | GGCCATGCACYWCYTCTC |  | 12 |
| Eukarya  (18S rRNA gene) | Euk1209F | CAGGTCTGTGATGCCC | 183 | 13 |
|  | Euk1392R | ACGGGCGGTGTGTRC |  | 14 |
| Fungi  (ITS region) | ITS1F | TCCGTAGGTGAACCTGCGG | Varied | 15 |
|  | ITS4R | TCCTCCGCTTATTGATATGC |  |  |

References:

1. Suryavanshi, M. V. *et al.* Hyperoxaluria leads to dysbiosis and drives selective enrichment of oxalate metabolizing bacterial species in recurrent kidney stone endures. *Sci. Rep.* **6,** 34712; 10.1038/srep34712 (2016).

2. Klocke, M. *et al.* Monitoring Lactobacillus plantarum in grass silages with the aid of 16S rDNA-based quantitative real-time PCR assays. *Syst. Appl. Microbiol.* **29,** 49–58 (2006).

3. Frank, D. N., Wilson, S. S., St Amand, A. L. & Pace, N. R. Culture-independent microbiological analysis of foley urinary catheter biofilms. *PLoS One* **4,** e7811; https://doi.org/10.1371/journal.pone.0007811 (2009).

4. Suryavanshi, M. V., Bhute, S. S., Bharti, N., Pawar, K. & Shouche, Y. S. Eubacterial diversity and oxalate metabolizing bacterial species (OMBS) reflect oxalate metabolism potential in Odontotermes gut. *J. Pure Appl. Microbiol.* **10**, 2035-2044 (2016).

5. Caporaso, J. G. *et al.* QIIME allows analysis of high-throughput community sequencing data Intensity normalization improves color calling in SOLiD sequencing. *Nat. Methods.* **7,** 335–336 (2010).

6. Langille, M. G. I. *et al.* Predictive functional profiling of microbial communities using 16S rRNA marker gene sequences. *Nat. Biotechnol.* **31,** 814–21 (2013).

7. Parks, D. H. & Beiko, R. G. Identifying biologically relevant differences between metagenomic communities. *Bioinformatics* **26,** 715–21 (2010).

8. The R Core Team.  R: A Language and Environment for Statistical Computing. Vienna, Austria : the R Foundation for Statistical Computing. ISBN: 3-900051-07-0. Available online at [http://www.R-project.org/](http://www.r-project.org/). (2013).

9. Khammar, N. *et al.* Use of the frc gene as a molecular marker to characterize oxalate-oxidizing bacterial abundance and diversity structure in soil. *J. Microbiol. Methods* **76,** 120–7 (2009).

10. Vital, M. *et al.* A gene-targeted approach to investigate the intestinal butyrate-producing bacterial community. *Microbiome* **1,** 8; https://doi.org/10.1186/2049-2618-1-8 (2013).

11. Gittel, A., Sorensen, K. B., Skovhus, T. L., Ingvorsen, K. & Schramm, A. Prokaryotic Community Structure and Sulfate Reducer Activity in Water from High-Temperature Oil Reservoirs with and without Nitrate Treatment. *Appl. Environ. Microbiol.* **75,** 7086–96 (2009).

12. Gantner, S., Andersson, A. F., Alonso-Sáez, L. & Bertilsson, S. Novel primers for 16S rRNA-based archaeal community analyses in environmental samples. *J. Microbiol. Methods* **84,** 12–8 (2011).

13. Giovannoni, S. J., DeLong, E. F., Olsen, G. J. & Pace, N. R. Phylogenetic group-specific oligodeoxynucleotide probes for identification of single microbial cells. *J. Bacteriol.* **170,** 720–26 (1988).

14. Lane, D. J. *et al.* Rapid determination of 16S ribosomal RNA sequences for phylogenetic analyses. *Proc. Natl. Acad. Sci.* **82,** 6955–59 (1985).

15. Borneman, J. & Hartin, R. J. PCR primers that amplify fungal rRNA genes from environmental samples. *Appl. Environ. Microbiol.* **66,** 4356–60 (2000).
